# Programmable Nanoscale Motion via Molecular Patterning on DNA Origami

**DOI:** 10.1002/anie.202523921

**Published:** 2026-01-16

**Authors:** Lars Paffen, Maurik Engelbert van Bevervoorde, Andoni Rodriguez‐Abetxuko, Loai Abdelmohsen, Remco van der Hofstad, Jan C.M. van Hest, Tania Patiño Padial

**Affiliations:** ^1^ Department of Biomedical Engineering and Chemical Engineering and Chemistry Institute for Complex Molecular Systems Eindhoven University of Technology Helix, P. O. Box 513 Eindhoven 5600 MB The Netherlands; ^2^ Department of Mathematics and Computer Science Institute for Complex Molecular Systems Eindhoven University of Technology Metaforum, P. O. Box 513 Eindhoven 5600 MB The Netherlands

**Keywords:** DNA origami, Enzyme kinetics, Nanomotors, Single‐particle tracking

## Abstract

Asymmetry in enzymatically driven nanomotor design, both structural and functional, is widely considered essential for propulsion. However, the interplay between particle geometry, enzyme distribution, and catalytic loading remains poorly defined, largely due to limited control over enzyme positioning that hinders quantitative analysis. Using DNA origami nanorods, we achieve precise spatial placement of urease enzymes with independently tunable coverage and asymmetry. Single‐particle tracking reveals that motility arises not solely from enzyme number or spatial arrangement but from a balance between catalytic loading and geometric anisotropy. Unexpectedly, maximal propulsion occurs at ∼25% urease end‐coverage, significantly below the conventional 50% end‐coverage, where half of the available binding positions on one structural half of the origami are occupied. Boundary Element Method simulations incorporating identical spatial parameters reproduce these findings, confirming that programmable enzyme patterning dictates diffusiophoretic propulsion. These results provide a quantitative framework linking topology, catalytic activity, and motion, revealing that optimal motility does not coincide with maximal asymmetry and advancing the rational design of enzyme‐powered DNA nanomotors.

## Graphical abstract

DNA origami nanorods enable precise urease placement with tunable coverage and asymmetry, revealing that propulsion depends on a balance of catalytic loading and geometry. Maximum speed occurs at ∼25% end‐coverage and declines as enzyme distribution becomes more symmetrical. Experiments and simulations establish a framework for rational nanomotor design using programmable enzyme patterning.

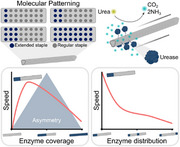

## Introduction

Motility plays an essential role in biology, where converting chemical energy into mechanical work drives complex functions such as molecular transport or cell motility.^[^ ^1^, ^2^ ^]^ Inspired by natural motor systems, in situ chemical reactions have been used to build artificial nanomotors that generate motion through substrate decomposition into products.^[^ ^3^, ^4^, ^5^, ^6^ ^]^ Among various propulsion strategies, enzyme‐powered nanomotors offer distinct advantages due to their biocompatibility and operation under mild conditions. Enzymes such as urease catalyze the decomposition of urea into ammonia and carbon dioxide, generating local chemical gradients that induce motion via diffusiophoresis.^[^ ^7^, ^8^, ^9^ ^]^ These systems have been proposed for applications including drug delivery,^[^ ^10^ ^]^ bio‐sensing,^[^ ^11^ ^]^ and the construction of active nanoscale assemblies.^[^ ^12^ ^]^

Directional propulsion requires symmetry breaking, typically achieved through asymmetry in particle geometry or in the spatial arrangement of catalytic sites.^[^ ^13^ ^]^ In Janus particles, asymmetry in structure or catalytic functionalization supports diffusiophoretic motion.^[^ ^14^, ^15^ ^]^ However, in most enzyme‐powered systems, the spatial arrangement of catalytic units is stochastic, making quantitative correlations between enzyme placement and motility difficult.^[^ ^16^ ^]^ At the nanoscale, achieving precise control over enzyme placement, loading, and orientation remains challenging, which has hindered mechanistic understanding. As a result, models developed for spherical Janus systems, where 50% catalytic end‐coverage is often assumed to yield maximal propulsion, remain largely untested for anisotropic geometries. Many enzyme‐driven nanomotor designs still lack precise control over topology, particle shape, the number of attached enzymes, or their spatial distribution.^[^ ^4^, ^16^ ^]^ As a result, it remains poorly understood how enzyme number and distribution govern motility.

DNA origami offers a unique platform to overcome these limitations. Through programmable self‐assembly, DNA nanostructures enable nanometer‐scale control over geometry and functionalization.^[^ ^17^, ^18^, ^19^ ^]^ This precision allows systematic variation of enzyme loading and spatial patterning, facilitating quantitative studies of how spatial asymmetry governs motion. Although DNA origami has been extensively used to construct static^[^ ^20^ ^]^ and dynamic^[^ ^21^ ^]^ nanodevices, its application in active, enzyme‐powered motile systems remains underexplored.

Here, we introduce a DNA‐origami nanorod platform that enables positioning of urease enzymes with tunable loading and spatial patterns. By varying the degree of asymmetry, we investigate how enzyme number and distribution influences propulsion behavior. Our results reveal that these rod‐like structures, with varying degrees of enzyme loading and patterning, deviate from the canonical Janus expectation (derived for spherical particles) that maximal propulsion occurs at ∼50% catalytic end‐coverage. In our nanorod system, optimal motion instead arises from a balance between catalytic loading and spatial asymmetry, rather than from maximal asymmetry alone. Boundary Element Method simulations of phoretic slip velocities corroborate these findings (see the Model and Simulation Section of the Supporting Information). Together, these results establish a quantitative framework linking enzyme topology, catalytic activity and motility, providing design principles for the rational design of enzyme‐powered nanomachines.

## Results and Discussion

**Figure 1:**
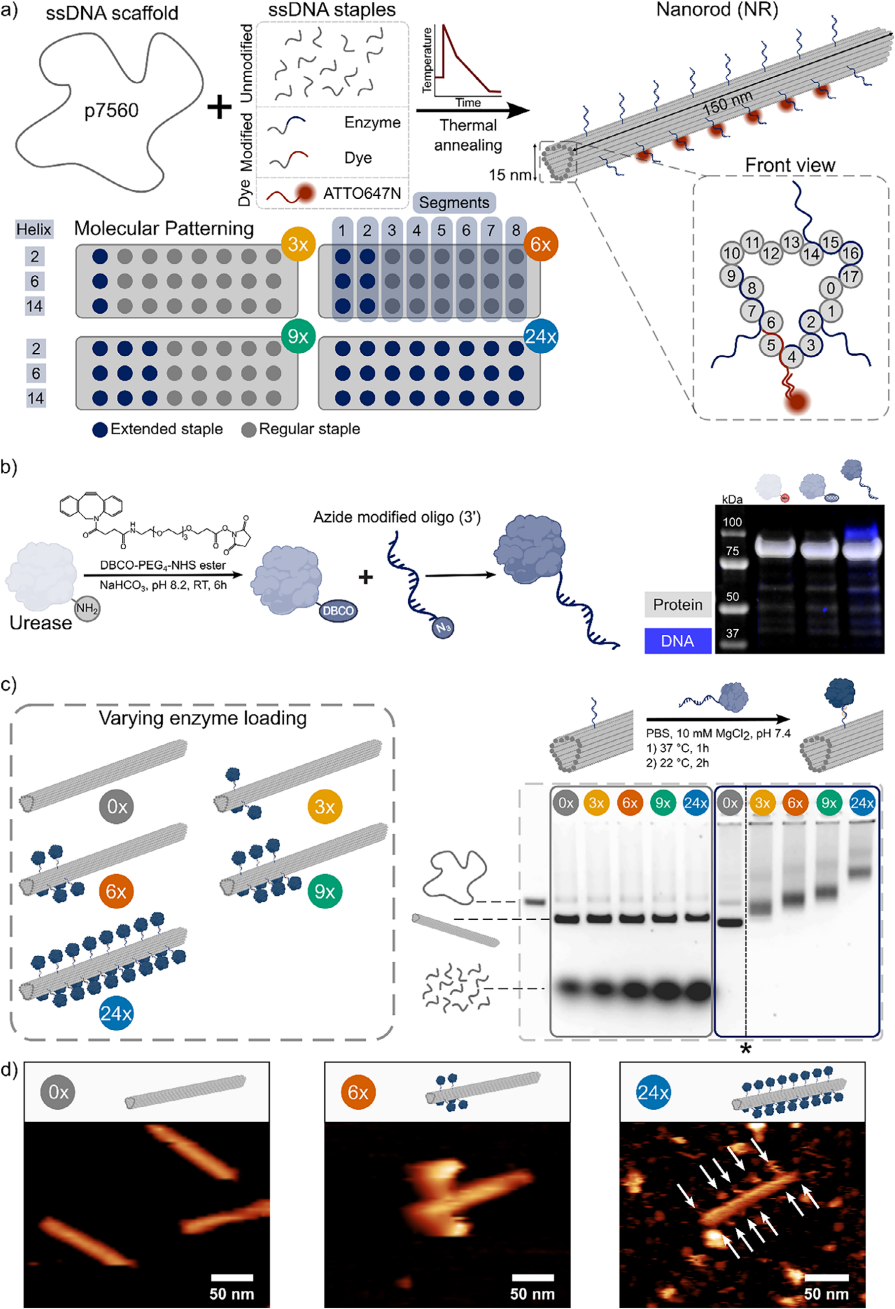
Formation and characterization of urease‐functionalized DNA nanorods. a) Schematic of DNA nanorod (NR) self‐assembly with 24 radially distributed handle strands extending from selected helices for urease attachment; four valency designs (3x, 6x, 9x, 24x) were used with urease functionalization on defined helices (horizontal) and segments (vertical). b) Urease‐oligo conjugation via NHS ester coupling of a DBCO handle, followed by SPAAC with azide‐modified ssD‐NA; SDS‐PAGE stained with Coomassie and SYBR Gold confirms conjugation (lanes 1–3: native urease, urease‐DBCO, urease‐oligo). c) Graphical representation of the urease‐functionalized NRs. Functionalized NRs were formed by incubation with urease‐oligo conjugates; Agarose Gel Electrophoresis (AGE) confirms NR folding (left) and stepwise size increase with higher valency (right). Uncropped and unspliced gel (*) is provided in the Supporting Information (Figure S3,S4). d) AFM topography of NRs with 0x, 6x, and 24x urease loaded, confirming proper urease localization.

In order to investigate the influence of spatial and numerical control of enzymes on nanoparticle motility, we employed an 18‐helix DNA origami bundle design (150x15 nm) that we refer to as nanorod (NR).^[^ ^22^, ^23^ ^]^ This structure enabled site‐specific functionalization via 24 radially distributed extended handle strands (Tables S1–S3), allowing precise attachment of enzyme–oligonucleotide conjugates. Additionally, six handle strands complementary to ATTO647N‐modified oligonucleotides (Tables S1,S2,S4) were incorporated to facilitate fluorescent labeling for single‐particle tracking fluorescence microscopy (SPT‐FM). We designed four NR constructs with increasing enzyme valency (3x, 6x, 9x, and 24x) and assembled them via thermal annealing (Figure 1a; Tables S1,S2,S5). To enable enzyme conjugation, urease was functionalized with a complementary oligonucleotide through NHS ester coupling followed by a strain‐promoted azide‐alkyne cycloaddition (SPAAC) with an oligonucleotide (Table S3). Successful conjugation of ssDNA to urease was confirmed by reducing SDS–PAGE, which revealed an increase in molecular weight from ∼90 kDa to ∼100 kDa (MW_Subunit_: ∼91 kDa; MW_oligo_: 6.6 kDa) and a co‐localized fluorescent signal from DNA staining, indicative of oligonucleotide (oligo) attachment (Figure 1b). Quantification of the degree of functionalization (DoF) of reduced urease subunits suggested ∼11% labeling efficiency, corresponding to an approximate 0.7:1 oligo‐to‐enzyme ratio for the hexameric protein (Figure S2). Assembly of the urease‐functionalized NR constructs was achieved by incubating the folded NRs with urease–oligo conjugates, allowing for hybridization to the extended handle strands. Agarose gel electrophoresis (AGE) confirmed successful conjugation, showing a discrete stepwise increase in NR size with increasing enzyme valency (Figure 1c). Next, we employed atomic force microscopy (AFM) to resolve spatial placement of the enzymes and characterize the topography of the functionalized NRs. AFM imaging revealed distinct protein patterns localized near the NR surface in both the 6x and 24x constructs, confirming site‐specific urease placement at the intended positions (Figure 1d). To confirm that urease retained its catalytic activity following oligonucleotide functionalization and subsequent immobilization onto the NR, we performed a colorimetric urease activity assay based on the pH‐dependent absorbance shift of Phenol Red, measured at 560 nm (Figure S5 and Table S6). Kinetic parameters including the turnover number (*k_cat_*) and Michaelis‐Menten constant (K_M_) were determined for four enzyme states: native urease, intermediate conjugates (urease–DBCO and urease–oligo), and urease–oligo conjugates hybridized to the NR. To enable direct comparison across conditions, the relative catalytic efficiency (*k_cat_*/K_M_) was calculated for each state. Kinetic analysis revealed that urease retained most of its activity after DBCO and oligonucleotide conjugation, with relative catalytic efficiencies of 87% and 85%, respectively. Interestingly, immobilization on the NR increased catalytic efficiency to 145% relative to native urease, driven by a higher apparent *k_cat_* and slightly reduced K_M_. This enhancement likely arises from the formation of a urea‐rich hydration layer around the NR, driven by electrostatic interactions that locally increase substrate concentration.^[^ ^24^, ^25^ ^]^ Together, these findings highlight the potential of DNA origami NRs to not only preserve but also improve enzyme performance while enabling precise spatial control of enzyme localization for fundamental motility studies.

**Figure 2:**
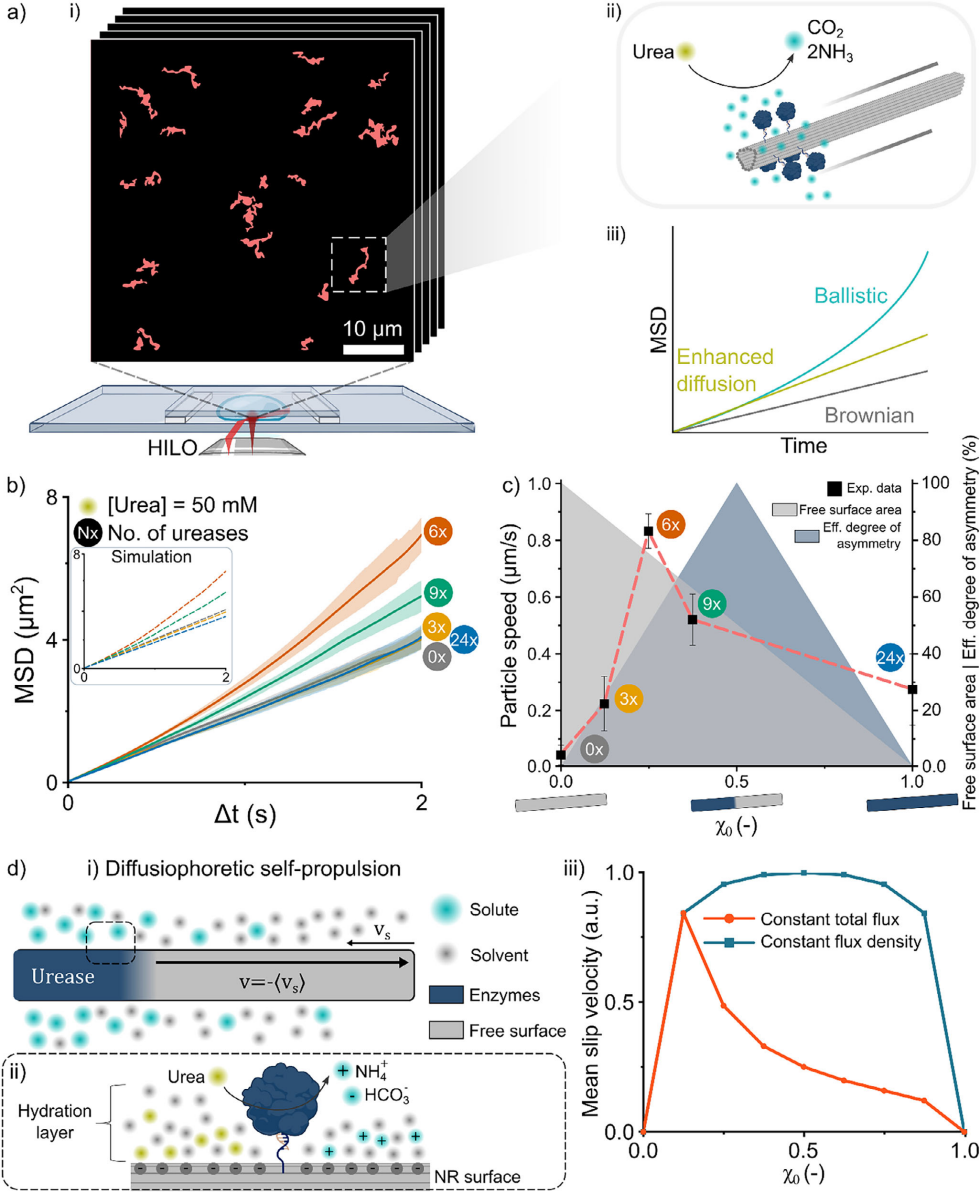
Single‐Particle Tracking of DNA nanorods with variable enzyme loading. a) Schematic overview of the experimental setup. (i) HILO illumination using a 640 nm inclined laser enables tracking of particles in solution while minimizing background noise. (ii) Illustration of urease‐induced nanorod propulsion via diffusiophoresis. (iii) Representative MSD profiles of particles exhibiting Brownian motion (grey), enhanced diffusion (yellow) and ballistic motion (cyan) (b) MSD profiles of urease‐functionalized nanorods with increasing enzyme valency (0x, 3x, 6x, 9x, 24x), showing enhanced motion for 6x and 9x designs. Retrieved MDSs from BEM simulation are shown as dashed lines (inset). Shaded areas represent ± SEM (Standard Error of the Mean). N > 180 particles for all conditions c) Particle speed plotted against the degree of functionalization (χ_0_). Free surface area on the NRs (grey) and effective degree of asymmetry (blue) are displayed as colored areas. Error bars represent SEM. d) Principles of diffusiophoretic self‐propulsion. (i) Local and asymmetric production of solutes creates a concentration gradient along the NR surface resulting in a diffusiophoretic slip flow with velocity (versus), and a corresponding net particle velocity (*v*). (ii) Zoom‐in on a single immobilized urease on the highly negatively charged NR surface, showing the hydration layer that facilitates both substrate (urea) as well as product (ammonium) accumulation. (iii) BEM simulated mean slip velocity under two conditions: (1) constant total flux and (2) constant flux density.

To study the motile behavior of our DNA origami NRs, we conducted a series of experiments based on Single‐Particle Tracking (SPT). SPT is widely used for real‐time visualization of biomolecules or nanoparticles, particularly in cellular environments.^[^ ^26^, ^27^ ^]^ In previous work, we successfully applied SPT to track antibody‐functionalized DNA origami structures, both in solution and on cell surfaces.^[^ ^28^ ^]^ SPT was performed using a super‐resolution microscope (Oxford NanoImager; ONI) equipped with software for real‐time particle tracking (Figure 2a(i)). Initial attempts using water as the medium resulted in rapid out‐of‐focus motion. Therefore, we switched to a more viscous solution (60% (v/v) glycerol) to slow down particle movement and enhance tracking accuracy.^[^ ^29^, ^30^ ^]^ While SPT is often combined with Total Internal Reflection Fluorescence (TIRF) microscopy, which excites fluorophores near the glass surface via a shallow evanescent field,^[^ ^31^ ^]^ we employed Highly Inclined and Laminated Optical sheet (HILO) illumination. HILO uses a less inclined laser angle, generating an evanescent field that penetrates deeper into the sample, allowing tracking of particles in solution while maintaining low background noise. For each condition, ten videos were recorded (1000 frames per video; 10 ms exposure; 100 fps), exciting the ATTO647N dye on the NRs with a 640 nm laser and thereby collecting trajectories of >100 particles per condition (Figure 2a(i)). By varying the degree of NR functionalization with urease while keeping the urea concentration constant (50 mM), we observed differences in mean squared displacement (MSD) as a function of enzyme valency and distribution across the surface. The enzymatic conversion of urea into CO_2_ and NH_3_ creates a local concentration gradient, highest near the urease sites, which drives particle motion toward lower concentration regions, a phenomenon known as self‐diffusiophoresis (Figure 2a(ii)).^[^ ^32^, ^33^ ^]^ Particle propulsion is reflected in a quadratic MSD profile when the motion is ballistic, in contrast to the linear MSD growth observed in both Brownian motion and enhanced diffusion, where the latter exhibits a higher slope of the MSD profile due to active processes (Figure 2a(iii)).^[^ ^34^ ^]^

In total, five urease‐functionalized NR designs were evaluated, including a non‐functionalized control. Following trajectory reconstruction from time‐resolved particle positions, the MSD was calculated for each condition and plotted over a 2‐s interval (Figure 2b). The MSD profiles of the 0x, 3x, and 24x urease‐functionalized NRs showed substantial overlap and followed a near‐linear trend, consistent with standard Brownian diffusion. In contrast, the 6x and 9x designs exhibited MSD profiles with slight deviations from linearity and significantly steeper slopes compared to the 0x, 3x, and 24x designs, suggesting the onset of active contributions beyond pure Brownian diffusion, though not indicative of fully ballistic behavior. By fitting the MSD to the full equation (Equation 1):^[^ ^32^ ^]^

(1) $$MSD=4D_{T}t+\frac{v^{2}}{2D_{R}^{2}}\left(2D_{R}t+e^{-2D_{R}t}-1\right)\;$$

motion parameters including the translational diffusion coefficient (*D_T_*), rotational diffusion coefficient (*D_R_*), and the particle speed (*v*) were extracted. The particle speed (*v*) was plotted as a function of the degree of functionalization (*χ_0_*), a parameter introduced in this work, representing the ratio of occupied (*N_occupied_*) over total enzyme positions (*N_total_*; Equation 2).

(2) $$\chi_{0}=\frac{N_{\mathrm{occupied}}}{N_{\mathrm{total}}}\;$$

With *N_total_* = 24, *χ_0_* ranges from 0 (non‐functionalized, 0 ureases) to 1 (fully functionalized, 24 ureases). To further support our experimental findings, we performed boundary element method (BEM) simulations using the retrieved motion parameters to generate simulated MSD profiles (Figure 2b). In the BEM, we solve for the product particle concentration gradient tangent to the micromotor surface, as this gradient induces a slip velocity that leads to propulsion.^[^ ^35^ ^]^ Averaging out these slip velocities over the micromotor surface gives the net propulsion speed, which we use together with sampled Brownian motion to simulate MSD trajectories (see the Model and Simulation Section of the Supporting Information). These simulations closely match the experimental MSDs, confirming the reliability of the extracted parameters and providing additional insight into nanomotor characteristics such as phoretic mobility (Figure S6). Alongside particle speed, the fraction of unfunctionalized (free) surface area and the degree of asymmetry were also plotted as a function of *χ_0_* (Figure 2c). Two measures were used to quantitatively describe the degree of symmetry (*φ*; Equation 3) and the effective degree of asymmetry (Equation 4).

(3) $$\varphi =1-\frac{L_{NR}-l_{E-E}}{L_{NR}-N_{E}}$$

Here, a 2D rod is dissected into 8 equally sized parts (referred to as “segments”), related to the positions on the physical rod to which the ureases are radially localized. In this equation, *L_NR_* resembles the total rod length (8 segments), *l_E‐E_* the maximum enzyme‐to‐enzyme distance (in segments) including the enzyme‐functionalized segments, and *N_E_* the total number of occupied enzyme segments (ranging from 1 to 8). The degree of symmetry (*φ*) will be further elaborated on when discussing the displacement of ureases over the NR at a constant enzyme loading (Figure 4), but it is a key parameter to determine the *effective degree of asymmetry* discussed in this section.

(4) $$\mathrm{Effective}\;\mathrm{degree}\;\mathrm{of}\;\mathrm{asymmetry}=\frac{N_{m}}{N_{H}}\;\times \left(1-\varphi \right),$$

Here, *N_m_* represents the number of segments on one half of the rod that lack their center‐symmetric analog on the other half of the rod. *N_H_* corresponds to the maximum number of enzyme segments on one half of the NR (*N_H_* = 4). Graphical representation of the metrics in Equations (3) and (4) is provided in Figure S7.

Nanoparticles functionalized on only one half (*χ_0_* = 0.5), like Janus particles, e.g., are commonly employed to induce structural asymmetry and, consequently, motility.^[^ ^36^, ^37^ ^]^ This design principle is supported by theoretical and mechanistic studies, which show that fully asymmetric functionalization of spheres and slender rods yields optimal self‐propulsion.^[^ ^38^, ^39^, ^40^ ^]^ In our system, we indeed observed self‐propulsion as a function of *χ_0_*. However, contrary to the expected maximum at *χ_0_* = 0.5, the particle speed peaked at *χ_0_* ≈ 0.25 (Figure 2c). To rule out local substrate depletion at higher functionalization levels, we calculated the Damköhler number (*Da*) for a fully functionalized NR (24x urease) in 50 mM urea (Equation 5 and Table S7). Here, *r* denotes the reaction rate, *R* the hydrodynamic radius of the nanorod, *d* the diffusioncoefficient of urea and *c_sub_* the concentration of urea far from the nanorod.

(5) $$Da=\frac{rR}{dc_{\mathrm{sub}}}=1.7\times 10^{-4}$$

As *Da* ≪ 1, substrate diffusion outpaces the enzymatic reaction rate, indicating that local substrate depletion is unlikely.^[^ ^41^ ^]^ The asymmetric distribution of product molecules over the NR creates an imbalance between intermolecular interactions between the solutes and the nanoparticle surface, resulting in a diffusiophoretic flow with a characteristic slip velocity (versus), and a net particle velocity (*v*) in the opposite direction (Figure 2d(i)).^[^ ^42^ ^]^ Due to the high negative charge density of DNA origami, a hydration layer forms along the NR surface, promoting local accumulation of urea and enhancing the catalytic efficiency of immobilized ureases relative to free enzyme in solution (Table S6).^[^ ^24^ ^]^ At neutral pH, the enzymatic products exist primarily as ammonium (NH_4_^+^) and bicarbonate (HCO_3_^−^). Thus, in addition to the hydrophilic substrate urea, the even more hydrophilic and positively charged NH_4_^+^ accumulates in the hydration layer. At higher degrees of functionalization, this may lead to elevated local NH_4_^+^ concentrations (Figure 4d(ii)), potentially causing product inhibition and contributing to the observed decline in particle speed prior to full asymmetry (*χ_0_ *= 0.5).^[^ ^43^ ^]^ An alternative explanation involves charge screening by NH_4_^+^ accumulation, which reduces the zeta potential (*ζ*) of the NR and thereby diminishes its (diffusio)phoretic mobility (Figure S6a).^[^ ^44^ ^]^ It is important to note that experimentally confirming complete occupancy of all theoretically available urease binding sites on each nanorod remains challenging, despite the convincing AGE and AFM data (Figure 1c,d). However, given the incremental step in enzyme loading by at least three ureases over the different designs, together with the critical role of not only enzyme number but also spatial positioning, it is reasonable to assume that the theoretical functionalization is closely approximated and that differences in motile behavior can still be meaningfully assessed.

To further investigate the effect of increasing enzyme loading, we simulated particle speed under two scenarios: (1) constant total flux and (2) constant flux density. In scenario (1), additional enzymes do not increase total product generation but occupy surface area, implying reduced activity per enzyme. In scenario (2), each enzyme contributes equally to the total flux. When plotted against the degree of functionalization (*χ_0_*), scenario (1) yields a monotonic decrease in speed, while scenario (2) shows a peak at *χ_0_* = 0.5, consistent with theoretical predictions (Figure 2d(iii)). Comparison with experimental data (Figure 2c) reveals that the observed speed profile lies between these two extremes. This suggests that increasing enzyme density toward complete structural asymmetry does not proportionally enhance catalytic output, likely due to product inhibition and surface charge screening effects. Thus, optimizing nanomotor performance requires careful consideration of both the quantity and spatial arrangement of catalytic elements.

**Figure 3:**
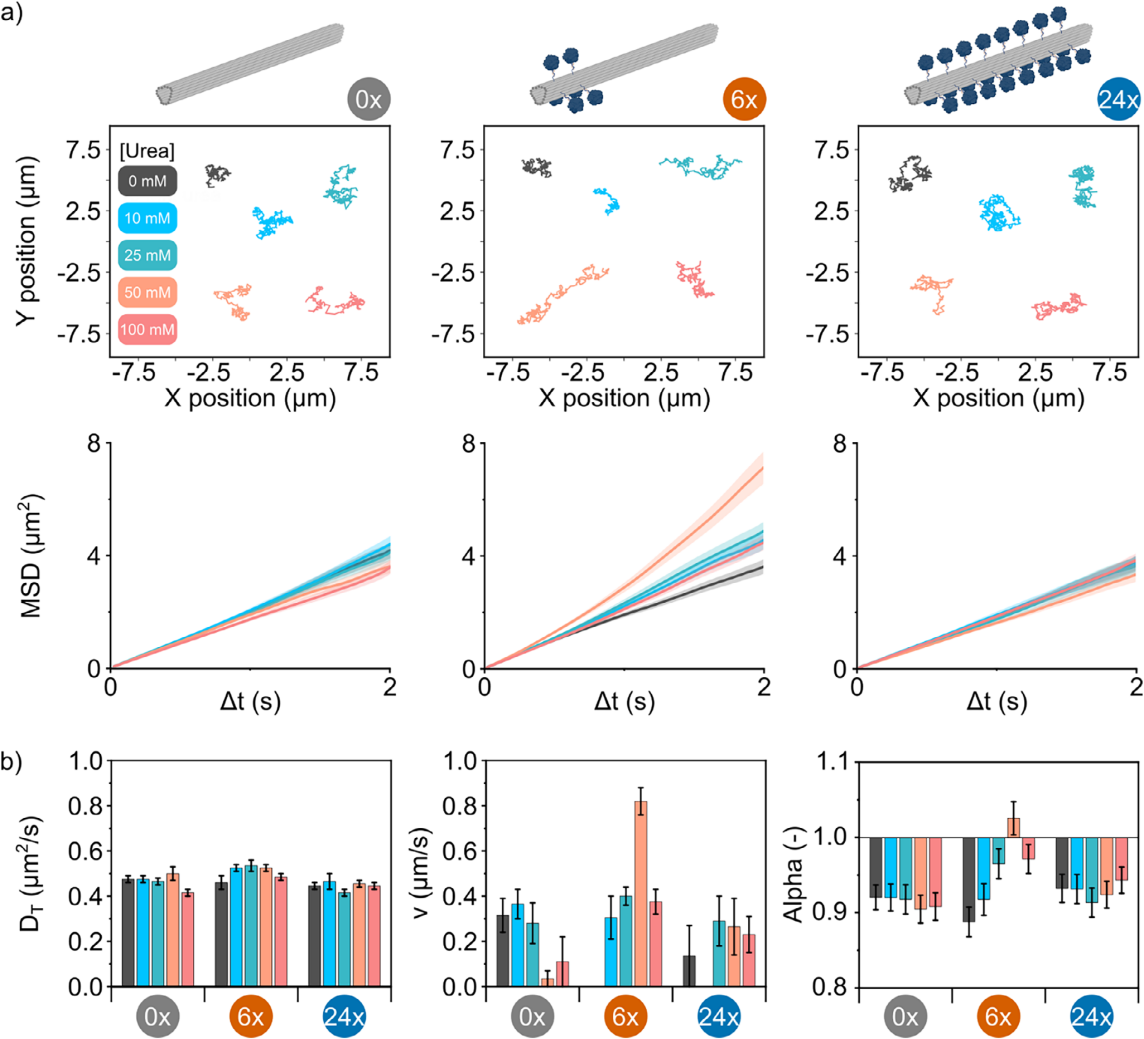
Motion analysis of DNA nanorods across increasing urea concentrations. a) Representative single‐particle trajectories (top) and corresponding MSD profiles (bottom) of 0x, 6x and 24x urease‐functionalized NRs. Shaded area represents ± SEM and n > 141 particles per condition. b) Bar graphs showing the translational diffusion coefficient (D_T_), particle speed (v) and global exponent of the MSD (α) extracted by fitting quadratic and logarithmic models to the MSDs, respectively. Error bars represent SEM and n > 141 particles per condition.

To elucidate the enhanced motility observed in asymmetrically urease‐functionalized NRs, we systematically investigated their motion behavior as a function of fuel concentration. Three distinct NR designs were evaluated: non‐functionalized (0x; *χ_0_* = 0), asymmetrically functionalized (6x; *χ_0_* = 0.25), and symmetrically functionalized (24x; *χ_0_* = 1). Each construct was analyzed across five urea concentrations (0, 10, 25, 50, and 100 mM) using single‐particle tracking (SPT) as previously described. The resulting trajectories were collected and analyzed to extract key motion parameters, including mean squared displacement (MSD), translational diffusion coefficient (*D_T_*), particle speed (*v*), and the global MSD exponent (*α*) (Figure 3). Representative trajectories and MSD profiles reveal that both 0x and 24x NRs exhibit negligible changes in motility across all tested urea concentrations. In contrast, 6x NRs display a clear transition from linear MSD behavior at 0 mM to a super‐diffusive regime at 50 mM, indicative of enhanced propulsion (Figure 3a). To further quantify this behavior, we assessed the MSD profiles on a logarithmic scale (Figure S8) and performed a logarithmic fit (Equation 6) to determine the global MSD exponent (*α*), which remained near or slightly above 1, consistent with enhanced diffusion rather than fully ballistic motion.^[^ ^45^ ^]^

(6) $$MSD=D_{T}\;t^{\alpha }$$

To extract the translational diffusion coefficient (*D_T_*) and propulsion speed (*v*), MSD data were fitted to the quadratic model (Equation 7).^[^ ^32^ ^]^

(7) $$\mathrm{MSD}=4D_{T}t+v^{2}t^{2}\;$$

The results reaffirm the trend observed in the MSD profiles (Figure 3b). While 0x and 24x NRs show no significant change in *D_T_* or *v* with increasing fuel concentration, 6x NRs exhibit a marked increase in particle speed from ∼0.0 µm/s at 0 mM to ∼0.8 µm/s at 50 mM urea, whereas the presence of 10 and 25 mM urea presents no significant increase with respect to the non‐functionalized NRs. At 100 mM, particle speed declined to ∼0.4 µm/s. We attribute this reduction at high fuel concentrations to environmental saturation with reaction products (CO_2_ and NH_3_). At elevated urea levels, the gradient between local product concentration near the NRs and the bulk environment diminishes, resulting in a more homogeneous distribution. This reduces the driving force for diffusiophoretic motion, thereby attenuating motility. Additionally, substrate inhibition at elevated urea concentrations may contribute to reduced motility.^[^ ^46^ ^]^

**Figure 4:**
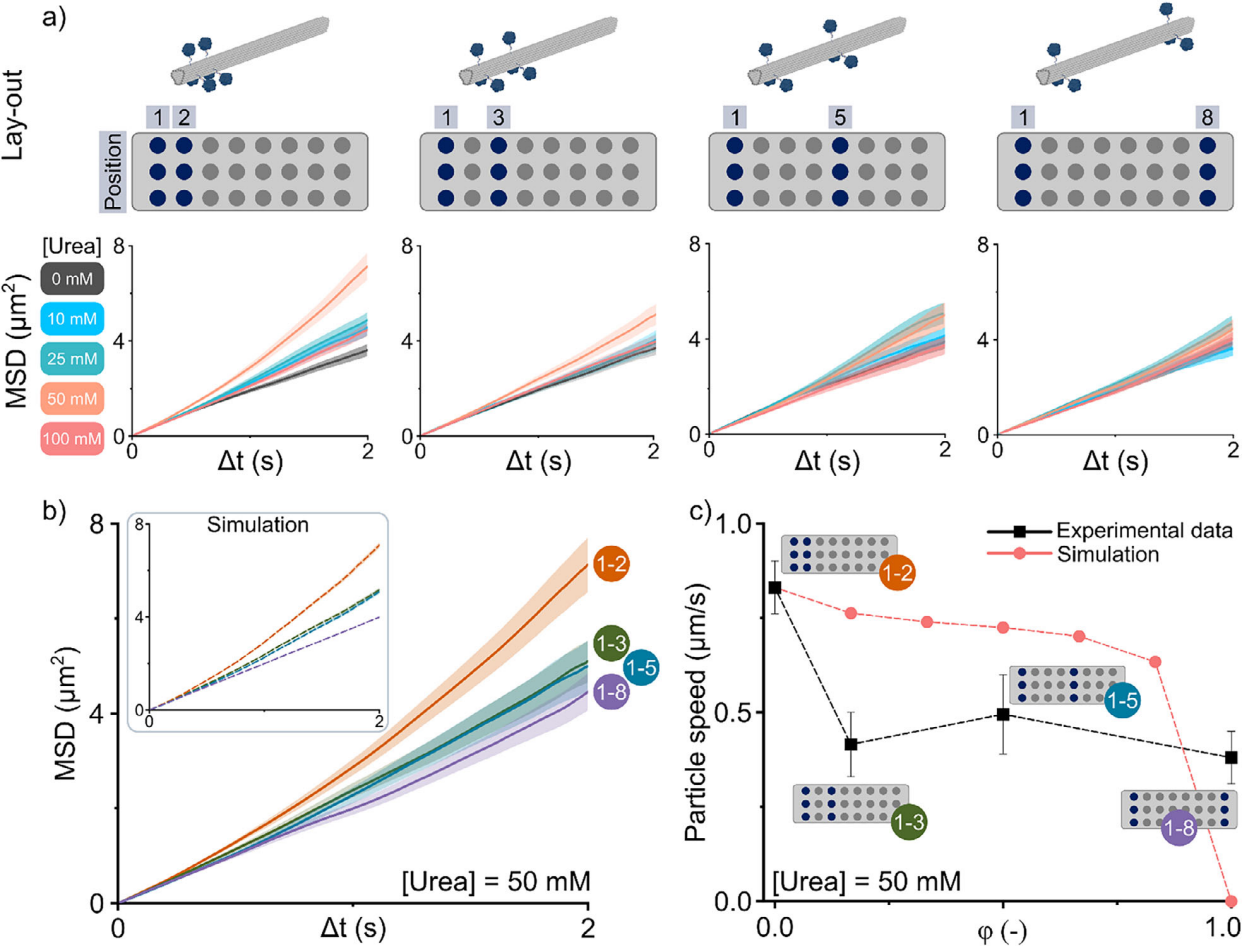
Effect of urease spatial arrangement on NR motility at constant enzyme loading. a) Graphical representation and lay‐out of NR designs with increasing symmetry: 1–2 (φ = 0), 1–3 (φ = 0.17), 1–5 (φ = 0.5) and 1–8 (φ = 1). Corresponding MSD profiles are shown for each design under varying urea concentrations. Shaded regions indicate SEM; n > 112 particles per condition. b) MSD profiles of the NR designs at 50 mM urea, including a BEM simulation (inset). c) Particle speed as a function of symmetry (φ) comparing experimental data (black) with simulated values (pink). Error bars represent SEM.

To isolate the effect of urease spatial distribution on NR motility, we employed the best‐performing asymmetric configuration (6x) as a benchmark. Rather than increasing the total enzyme count to achieve symmetry, we maintained a constant urease valency (six enzymes per NR) and systematically varied their spatial distribution to transition from a fully asymmetric (*φ* = 0) to a fully symmetric (*φ* = 1) configuration. The parameter *φ* quantifies the degree of symmetry based on the spacing between enzymes. Unlike the degree of functionalization (*χ_0_*) and the effective degree of asymmetry used earlier, *φ* changes solely through the repositioning of a fixed number of enzymes, without altering their total number (Equation 3). This closely relates to the simulated situation discussed before where we assumed constant total enzyme flux (Figure 2d(iii)). Starting from the original 6x design, in which three out of six ureases were radially positioned at the inner site (position 2), we progressively displaced these enzymes to distal positions (3, 5, and 8) by modifying the placement of the extended handle strands (Figure 4a). Following NR folding, purification, and urease‐oligo hybridization, SPT was performed across all four configurations under increasing urea concentrations, using the same protocol as for the 0x, 6x, and 24x designs shown earlier. Again, trajectory analysis and MSD profiling revealed that enhanced motility was most pronounced at 50 mM urea (Figure 4a). Therefore, the MSD profiles at this concentration were overlaid to facilitate direct comparison (Figure 4b). Fitting these profiles to Equation (1) enabled extraction of motion parameters, which were subsequently used in BEM simulations (see the Model and Simulation Section of the Supporting Information). The simulated MSDs closely matched experimental data, except for the fully symmetric design (positions 1–8), where the experimental MSD deviated from the expected linear behavior (Figure 4b). In general, a clear trend can be observed: increasing symmetry in urease distribution correlates with reduced motility. To quantify this relationship, we plotted both experimental and simulated particle speed as a function of *φ* (Figure 4c). We observe that experimentally retrieved speeds declined more sharply than simulated ones. A modest shift from full asymmetry (*φ* = 0; positions 1–2) to partial asymmetry (*φ* = 0.17; pos. 1–3) resulted in a reduction in particle speed of ∼50%. Further displacement of the enzymes toward higher degrees of symmetry (*φ* = 0.5; pos. 1–5) did not yield additional speed loss, although phoretic mobility clearly decreased (Figure S6b). Only at full symmetry (*φ* = 1; pos. 1–8), the particle speed dropped again but not to the extent predicted by simulations, which expected complete suppression of motion. However, since the simulated MSD profile of the fully symmetric design already indicated some deviations from the experimental profile (Figure 4b), it might be that the population of particles in case of the 1–8 configuration may not have been optimal. Nonetheless, these results demonstrate that motility is highly sensitive to the spatial arrangement of catalytic sites. A modest loss of asymmetry directly correlates with a reduction in active motion, underscoring the critical role of enzyme localization in designing motile nanostructures.

## Conclusion

We demonstrate that spatial and numerical enzyme patterning on DNA origami NRs enables programmable control over motility. By combining single‐particle tracking with spatially resolved simulations, integrating both experimental as well as theoretical tools, we quantitatively dissect the roles of asymmetry and catalytic loading in diffusiophoretic propulsion, providing mechanistic insights into enzyme‐driven motion. Our findings reveal that propulsion is governed by the interplay between enzyme distribution and geometric asymmetry, quantitatively linking particle topology, catalytic activity, and motion. Counterintuitively, the optimal functional anisotropy for efficient motion does not necessarily coincide with the highest effective degree of asymmetry. This is emphasized by the observed maximum propulsion speed at a degree of functionalization (*χ_0_* = 0.25), well below the asymmetric configuration (*χ_0_* = 0.5). The critical role of precise urease placement, and the resulting asymmetry, is further highlighted by the observed decline in particle speed as the enzyme spacing increases toward full symmetry (*φ* = 1). Since DNA origami offers accurate control over enzyme number and position, it serves as an ideal modular platform for constructing synthetic nanomotors with defined structural and functional features. This study presents the first systematic quantification of how catalytic loading and spatial asymmetry jointly dictate nanoscale propulsion, offering a framework for the rational design of enzyme‐powered nanomachines.

## Experimental Section

The data that support the findings of this study are available in the Supporting Information of this article.

## Supporting Information

The authors have cited additional references within the Supporting Information.^[^ ^35^, ^41^, ^47^, ^48^ ^]^

## Conflict of Interests

The authors declare no conflict of interest.

## Supporting information



Supporting Information

## Data Availability

The data that support the findings of this study are available from the corresponding author upon reasonable request.
