# Supplementary material for: Programmable Nanoscale Motion via Molecular Patterning on DNA Origami
Source: Angew Chem Int Ed Engl. 2026 Jan 16;65(8):e23921. doi: 10.1002/anie.202523921 (PMC12910145; doi:10.1002/anie.202523921)
Supplement: Supplementary file 1 — Supporting Information [file ANIE-65-e23921-s001.pdf]

Supporting Information

Programmable Nanoscale Motion via Molecular Patterning on DNA Origami

Lars Paffen[a], Maurik Engelbert van Bevervoorde[b], Andoni Rodriguez-Abetxuko[a], Loai Abdelmohsen[a], Remco van der Hofstad[b], Jan C.M. van Hest[a] and Tania Patiño Padial*[a]

[a] L. Paffen, A. Rodriguez-Abetxuko, L. Abdelmohsen, J. C. M. van Hest, T.P. Padial
Department of Biomedical Engineering and Chemical Engineering and Chemistry
Institute for Complex Molecular Systems, Eindhoven University of Technology
Helix, P. O. Box 513, Eindhoven, 5600 MB, the Netherlands
E-mail: t.patino.padial@tue.nl

[b] M. Engelbert van Bevervoorde, R. Van der Hofstad
Department of Mathematics and Computer Science
Institute for Complex Molecular Systems, Eindhoven University of Technology
Metaforum, P. O. Box 513, Eindhoven, 5600 MB, the Netherlands

Contents

Experimental Section 2
1. Materials 2
2. Methods 2
2.1 Nanorod Folding 2
2.2 Purification of NRs by PEG Precipitation 2
2.3 Urease-oligo conjugation 2
2.4 SDS-PAGE gel electrophoresis 3
2.5 NR – Antibody/Aptamer conjugation 3
2.6 Atomic Force Microscopy 3
2.7 Kinetic characterization of urease conjugates 3
2.8 Single Particle Tracking Experiments 4
2.9 Modeling equations and assumptions 4
3. Supplementary figures and tables 5
Figure S1 | Schematic of DNA nanorod (NR) self-assembly 5
Figure S2 | SDS-PAGE stained with Coomassie used for band intensity analysis 5
Figure S3 | Uncropped and unspliced agarose gel (fig. 1c) 6
Figure S4 | Zoom-in of the uncropped and unspliced agarose gel (fig. 1c) 7
Figure S5 | (a) Michaelis–Menten plot for determination of K_m (b) Kinetic analysis for determination of k_{cat}. 7
Figure S6 | Phoretic mobility b of NRs 8
Figure S7 | Graphical representation of the metrics used for Equations 3 and 4 8
Figure S8 | Log-Log plots of the MSD profiles shown in Fig. 2a 8
Table S1 | Handle-extended staple strands for urease immobilization on the 18-helix bundle DNA nanorod. 9
Table S2 | Sequences of unmodified staple strands of the 18-helix bundle DNA nanorod 10
Table S3 | Anti-handle for urease labeling 10
Table S4 | Fluorescently labeled imagers 10
Table S5 | Sequences of unmodified staple strands of the 18-helix bundle DNA nanorod. 11
Table S6 | Kinetic parameters illustrating the urease activity of the different stages in the functionalization process. 14
Table S7 | Parameters used for estimation of Damköhler number 15
4. References 16
Model and Simulation Section 17
1. Problem statement and solution approach 17
2. Boundary element method 19
2.1 Interior Laplace problem in 2D 19
2.2 Exterior Laplace problem in 2D 22
2.3 2D Exterior Rod 28
3. References 30

Experimental Section

1. Materials

All DNA staples strands and modified oligonucleotides (including azide- and ATTO647N-modifications) were purchased from Integrated DNA Technologies (IDT).

2. Methods

2.1 Nanorod Folding

DNA origami nanorods (NRs) were assembled by mixing 20 nM of the p7560 DNA scaffold (Tilibit Nanosystems) with a 5x molar excess (100 nM) of staple strands (Table S1/S2/S5) in folding buffer (5 mM Tris, 1 mM EDTA, 25 mM NaCl, 12 mM $MgCl_2$, pH 8.5). For single-particle tracking (SPT), six extended staple strands (Table S1) were introduced into the mixture (100 nM per strand) together with complimentary ATTO647N-modified DNA oligos (Table S4) in a 2-fold excess (200 nM per complimentary extended staple strand). For surface decoration with urease–oligo conjugates, 24 extended staple strands (100 nM each) were added to the staple pool. Folding mixtures were aliquoted into 50 µL fractions in 200 µL PCR tubes and thermally annealed in a thermocycler using the following protocol: 80°C for 15 min; 80→60°C at −1°C/min; 60→25°C at −1°C per 23 min 20 s (Fig. S1). After annealing, NRs were stored at 4°C or directly purified from excess staples for further processing.

2.2 Purification of NRs by PEG Precipitation

Folded NRs were purified from excess staples by polyethylene glycol (PEG) precipitation. All 50 µL aliquots were collected and transferred to a 1.5 mL DNA LoBind Eppendorf tube and gently mixed 1:1 (v/v) with 2X PEG Buffer (10 mM Tris, 2 mM EDTA, 1010 mM NaCl, 15% (w/v) PEG8000). Mixtures were incubated on ice for 10 min after which they were centrifuged at 21,000 x g for 25 min at 16°C. The supernatant was carefully removed using a pipette and the pellet was resuspended in storage buffer (1X PBS, 10 mM $MgCl_2$, pH 7.4) for 2-3 hours at 30°C and 350 rpm on a shaking incubator. NR concentration was determined by measuring the absorbance at 260 nm of a 2 µL droplet on a spectrophotometer (NanoDrop), using a molar extinction coefficient (ε) of 94,500,000 M-1·cm-1. Successful folding of the NRs was assessed by agarose gel-electrophoresis (AGE). Gel samples were prepared at a final NR concentration of 10 nM in storage buffer and 1.25% (w/v) Ficoll-400. The samples were loaded on a 1.5% (w/v) agarose gel (0.5X TBE, 10 mM $MgCl_2$, 1X Midori Green Advance). Gels were run at 75 V for 90 min in an ice bath, imaged on a gel imager (Amersham ImageQuant 800, Cytiva), and analyzed in ImageJ.

2.3 Urease-oligo conjugation

Lyophilized urease type IX from jack bean (Hexamer MW: ~ 544,620 Da; Subunit MW: 90,770 Da; Sigma-Aldrich) was weighed (~45 mg) into a 1.5 mL Eppendorf tube and dissolved in 1 mL of 100 mM sodium bicarbonate buffer (pH 8.2). Protein concentration was determined by measuring the absorbance at 280 nm using a NanoDrop spectrophotometer and a molar extinction coefficient of 322,365 $M^{-1}\cdot cm^{-1}$. For NHS ester functionalization, 900 µL of the urease solution was mixed with 100 µL DMSO containing DBCO-(PEG)4-NHS ester (ABCR) at an 8-fold molar excess relative to the urease. The mixture was incubated at room temperature (RT) while shaking at 350 rpm for 6 hours. The reaction was dialyzed overnight at 4°C against 1L 1X PBS using Spectra/Por 4 dialysis membrane tubing (12–14 kDa MWCO; Repligen) to remove DMSO and unreacted DBCO-(PEG)4 -NHS ester. The dialyzed urease–DBCO was concentrated and buffer-exchanged with Amicon Ultra centrifugal filters (100 kDa MWCO; Millipore) by centrifugation at 10,000 x g for 3 min per load, washing 5x with 1X PBS, and recovering by reverse spin at 1,000 x g for 2 min. The protein concentration was determined via spectrophotometry as described above. Urease–oligo conjugates were formed by strain-promoted azide–alkyne cycloaddition (SPAAC). Urease-DBCO was diluted to 10 µM in 1X PBS and reacted with a 10-fold molar excess of an azide-modified oligonucleotide (Table S3) overnight at 4°C. Following overnight incubation, unreacted azide-oligos were removed using the same centrifugal filtration protocol as described earlier. After 5 washing steps, the absorbance of the flow-through was measured at 260 nm to check for the presence of free DNA. If present, washing was repeated for 2-3 rounds after which the urease-oligo was recovered and the protein concentration was again determined as described earlier.

2.4 SDS-PAGE gel electrophoresis

Conjugation was verified by SDS–PAGE. For each condition (urease, urease-DBCO and urease-oligo), 5 µg of protein in 1X PBS buffer was mixed 3:1 with reducing Laemmli SDS sample buffer (ThermoScientific) and denatured at 95°C for 5 min. and 5 µl of protein ladder (Precision Plus Protein; Bio-Rad) were loaded onto a pre-cast 4–20% Mini-PROTEAN TGX gel (Bio-Rad). Gels were run in 1X Tris/Glycine/SDS buffer (25 mM Tris, 192 mM glycine, 0.1% (w/v) SDS, pH 8.3; Bio-Rad) at 150 V for 55 min and imaged using Bio-Safe Coomassie G-250 protein stain (Bio-Rad) and SYBR™ Gold Nucleic Acid Gel Stain (Invitrogen) on the Amersham ImageQuant 800 (Cytiva). Images were analyzed using ImageJ v1.53t.

2.5 NR – Antibody/Aptamer conjugation

PEG-purified NRs (15 nM) were incubated with urease–oligo conjugates at a 3-fold molar excess relative to the number of handles (extended staples) per NR. Incubations were performed for 1 h at 37°C followed by 2 h at 22°C in storage buffer (1X PBS, 10 mM MgCl2) while shaking at 350 rpm. The functionalized origami structures were analyzed on a 1.5% (w/v) agarose gel together with the gel samples pre- and post-PEG precipitation as described above.

2.6 Atomic Force Microscopy

Topographic images of the (functionalized) origami NRs were captured in tapping mode under liquid conditions using a Cypher ES Environmental atomic force microscope (Oxford Instruments). Cantilevers (BL-AC40TS; Olympus) with sharpened silicon tetrahedral tips, nominal spring constant 0.09 N/m, and resonance frequency in water ~25 kHz were used. Substrates were prepared by mounting round laser-cut mica sheets (~1 cm²; Ted Pella) on magnetic metal discs using conductive double-sided adhesive tabs. NR solutions were diluted to 2-5 nM in imaging buffer (10 mM Tris, 1 mM EDTA, 10 mM $MgCl_2$, pH 8.0), and 5 µL of this solution was deposited onto the freshly cleaved mica substrate. After a 30-second incubation, 50 µL of imaging buffer was added on the mica sheet. Prior to imaging, ~80 µL imaging buffer was applied to the cantilever and the stage was manually lowered until the droplets on both the cantilever and the mica fused and formed a meniscus. The objective correction collar was set to 2.0. Images (512 × 512 px) were acquired over areas of 5.0, 2.5, 1.0 and 0.5 µm² with scanning and feedback parameters optimized for each image. All images were analyzed and processed in Gwyddion v2.64.

2.7 Kinetic characterization of urease conjugates

The measurements of the apparent Michaelis-Menten constant (appKM) of free urease, urease-DBCO, urease-oligo, and urease-NR were performed in 96-well plates (final volume 200 µL), at 25 ºC, in phosphate buffered saline (10 mM phosphate, 137 mM NaCl, 2.7 mM KCl, pH 6.5). To measure the $^{(app)}K_M$ of the samples, we performed the catalytic reactions at growing concentrations of urea, from 0 to 100 mM, while keeping the assemblies and phenol red concentrations constant at 0.5 nM and 25 nM, respectively. The absorbance change of phenol red was monitored at 560 nm. The initial reaction rates were plotted against urea concentration and fitted to the Michaelis-Menten equation using nonlinear regression in GraphPad Prism 10 software. The extinction coefficient for phenol red at 560 nm (ε_{560nm} = 0.281 $mM^{-1}cm^{-1}$) was previously calculated varying ammonia concentration (from 0 to 1 mM) in the presence of 40 mM urea, 0.025 mM phenol red, PBS (1X pH 6.5) in 96-well plates. All measurements were performed in duplicate and blank controls (without enzyme) were included to account for background absorbance.

The turnover number ($^{(app)}k_{cat}$) of free urease, urease-DBCO, urease-oligo, and urease-NR was measured at a fixed concentration of urea and phenol red (50 mM and 25 nM, respectively) in phosphate buffered saline (10 mM phosphate, 137 mM NaCl, 2.7 mM KCl, pH 6.5). The concentrations of urea and phenol red were selected based on preliminary assays showing enzyme saturation conditions and a linear response. Reactions were performed at 25ºC in a 96-well plate (final volume per well of 200 µL) and the absorbance change of phenol red was monitored at 560 nm (ε_{560nm} = 0.281 $mM^{-1}cm^{-1}$). Each reaction was tested in a range of concentrations of urease. The initial reaction rates were plotted against urea concentration and fitted with a simple linear regression using GraphPad Prism 10 software. The reaction stoichiometry was considered during the conversion of absorbance measurements to the product formed. All measurements were performed in duplicate and blank controls (without enzyme) were included to account for background absorbance.

2.8 Single Particle Tracking Experiments

Glass cover slips (50x22 mm) were prepared by the following procedure: First, three labels for 1.5 mL tubes (Cryo-Babies, Diversiefied Biotech) were cut in half and two stacks of three cut labels were made. These two stacks were placed on the glass cover slip at ~1 cm from each edge. NRs were diluted to a final concentration of 1 nM in storage buffer (1X PBS, 10 mM MgCl2). Fresh urea stock solution (1 M) was prepared by dissolving ~60 mg urea (MW 60.06 g/mol; Sigma-Aldrich) in MQ water; working solutions of 500, 250, 100, and 0 mM were prepared from the stock. A 75% (v/v) glycerol solution was made by adding 250 µL MQ water to 750 µL glycerol. An 8 µL droplet of 75% (v/v) glycerol was placed between the label stacks. First, a 1 µL droplet of the 1 nM NR solution was added to this droplet and thoroughly mixed by pipetting up and down. Immediately before imaging, the slide was mounted and 1 µl of the desired urea solution (1 M, 500, 250, 100, or 0 mM) was added to the glycerol–NR droplet and thoroughly mixed after which a square glass cover slip (22x22 mm) was placed on top of the droplet being supported by the label stacks; SPT acquisition started immediately. Single-particle tracking (SPT) was performed on an Oxford NanoImager (ONI) equipped with a 100x oil-immersion objective. A 640 nm laser operating at 7.1 mW with an incident angle of 48.5°, enabling Highly Inclined and Laminated Optical (HILO) sheet measurements, was used to excite the fluorophores (ATTO647N) on the NRs. Per condition (NR design – fuel concentration combination) five 10-second videos (1,000 frames per video; 10 ms exposure; 100 fps) were recorded. Trajectories were generated in NimOS software. Here, the individual particle positions over all frames were transformed into trajectories using the following filter settings: max frame gap of 20, max. distance between frames of 0.8 µm, an exclusion radius of 1.2 µm and a minimum number of steps of 100. Track steps were exported and analyzed with the Python-based Nano-micromotor Analysis Tool (NMAT) v0.7 (https://github.com/rafamestre/NMAT-nanomicromotor-analysis-tool). Using this tool, motion characteristics like mean-squared displacement (MSD) and mean-squared angular displacement (MSAD) were retrieved, including logarithmic fits to determine the anomalous diffusion exponent α. Quadratic and full MSD fits (to Eq. 1) to obtain diffusion coefficients and particle speeds were performed using a SciPy function (https://docs.scipy.org/doc/scipy/reference/generated/scipy.optimize.curve_fit.html).

2.9 Modeling equations and assumptions

To simulate motility, we followed Popescu et al.'s model of self-diffusiophoresis.[1] Here, particle propulsion is driven by gradients in product concentration along the micromotor surface, following Eq. S1.

$$v_{\text{particle}} = -b\langle \nabla_{||} u \rangle \text{ (Eq. S1)}$$

In Eq. 3, u is the product concentration, b the phoretic mobility (which encodes interactions between product particles and the micromotor), and $\nabla_{||}$ the gradient tangent to the micromotor surface. The term $\langle \nabla_{||} u \rangle$ is then the concentration gradient tangent to the surface, averaged out over the surface. Key model assumptions are that there is no fuel depletion, the phoretic mobility is constant, and product concentration is significantly smaller than fuel concentration. Under these assumptions, we solve a boundary value problem in 2D for the product concentration at the micromotor surface, using the Boundary Element Method as described in Model and Simulation Section 2. The pseudocode in Algorithm 1 of Model and Simulation Section 2.3 shows how we incorporate Brownian motion into the propulsion model to simulate MSD trajectories.

3. Supplementary figures and tables

Figure S1 | Schematic of DNA nanorod (NR) self-assembly with 24 radially distributed handle strands extending from selected helices for urease attachment (blue) and 6 extended handle for the hybridization with a ATTO647N-modified oligonucleotide (red). Reference helix and nucleotide numbers are mentioned in the caDNAno template of the NR.

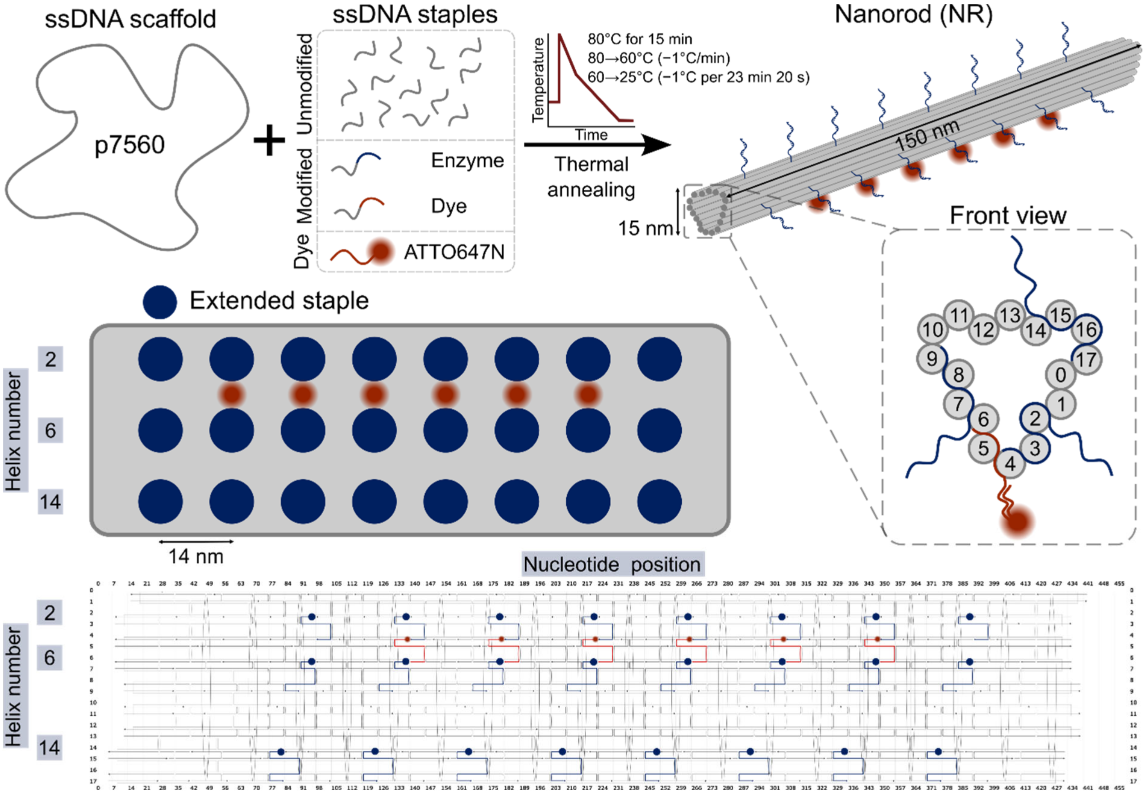

Figure S2 | SDS-PAGE stained with Coomassie used for band intensity analysis to determine the degree of labeling with the oligonucleotide (lanes 1–3: native urease, urease-DBCO, urease-oligo).

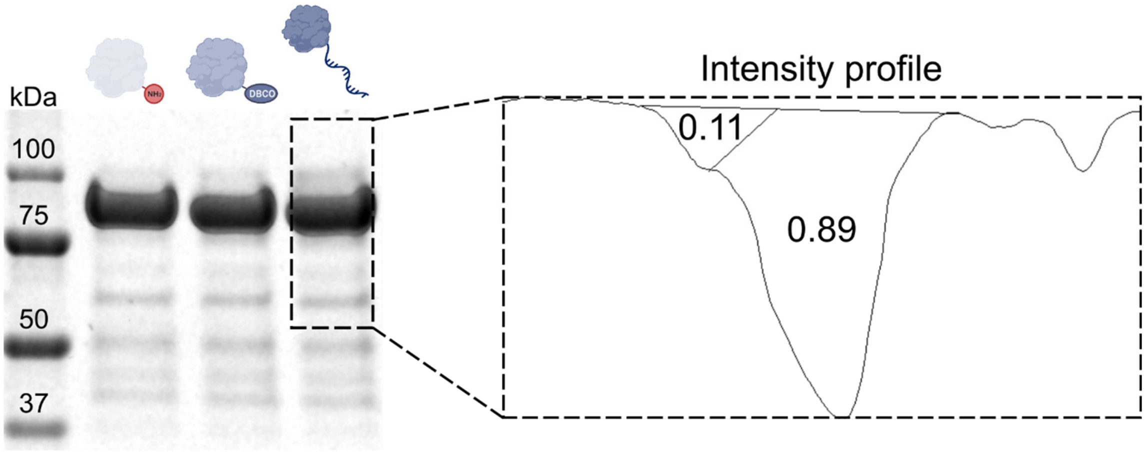

Figure S3 | Uncropped and unspliced agarose gel (fig. 1c) showing scaffold and the folded NR before purification and after functionalization with catalase- and urease-oligo, respectively.

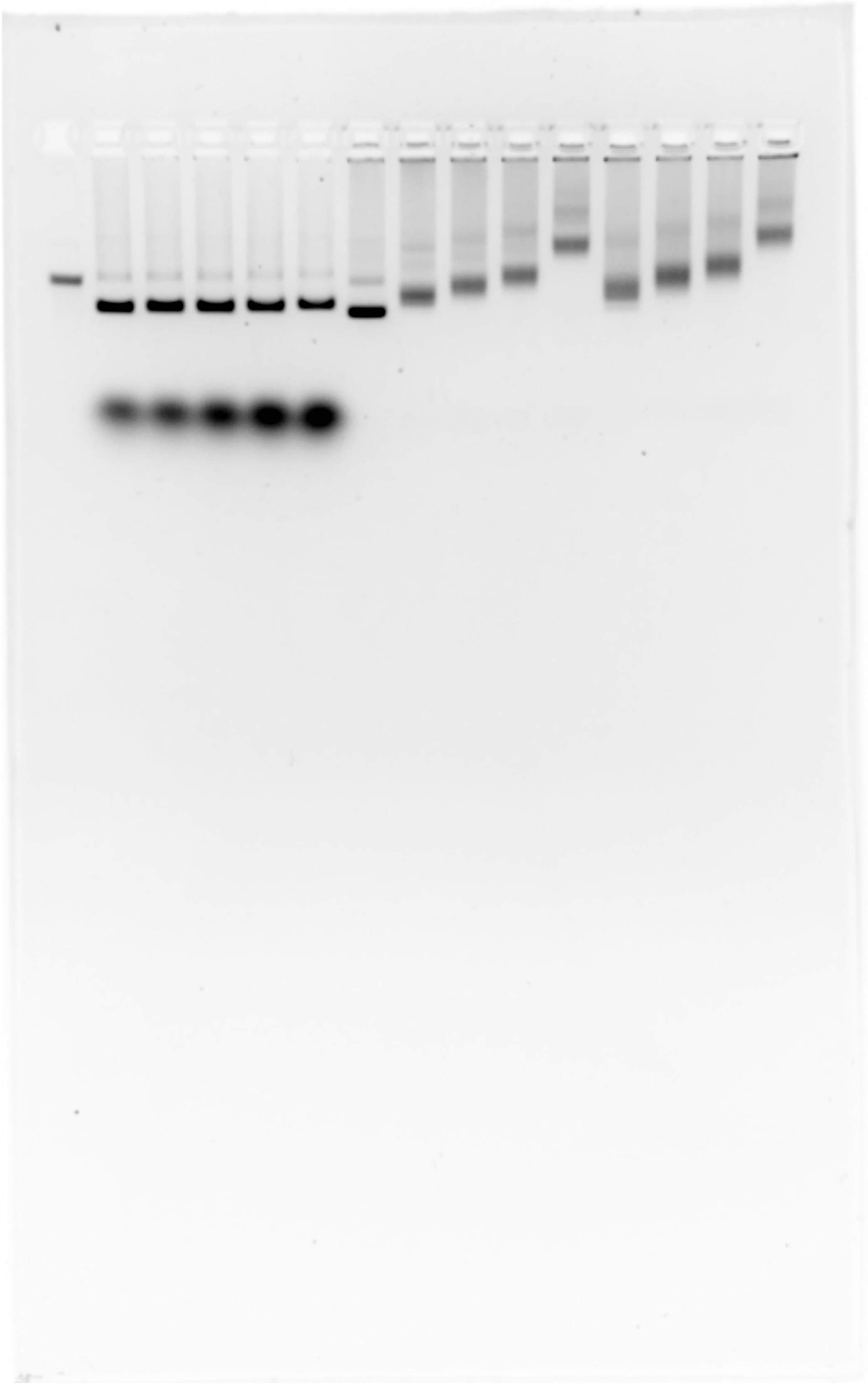

Figure S4 | Zoom-in of the uncropped and unspliced agarose gel (fig. 1c) showing scaffold and the folded NR before purification and after functionalization with catalase- and urease-oligo, respectively. The lanes with catalase functionalized NRs (3x, 6x, 9x, 24x) were sliced out to create the final gel in Figure 1C in the main text.

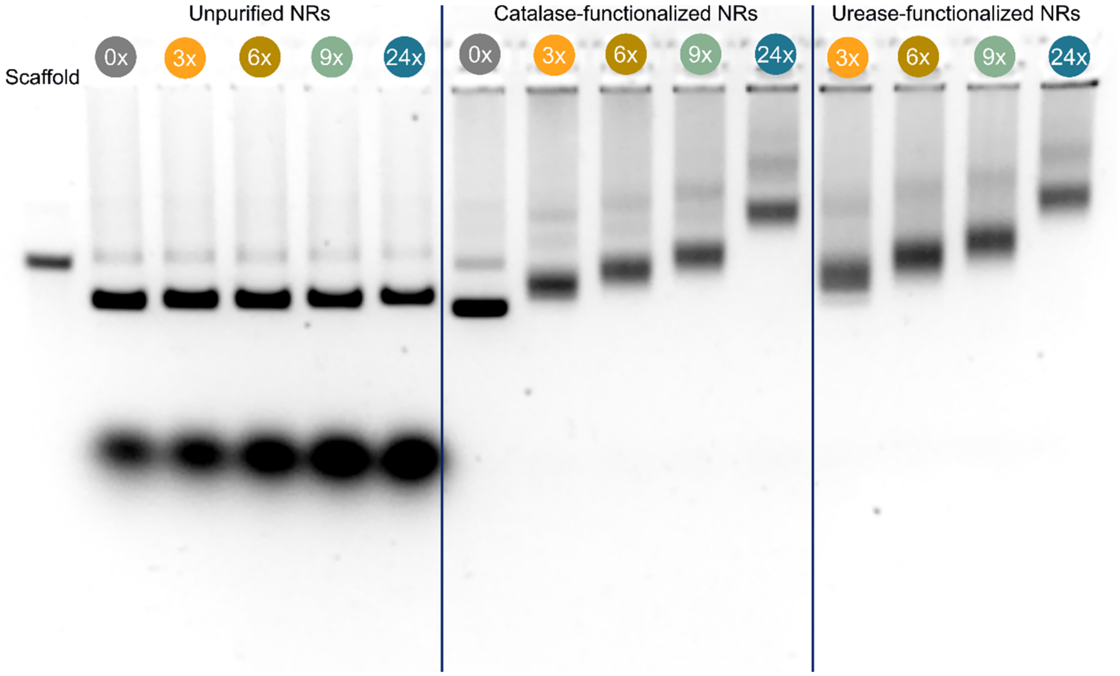

Figure S5 | (a) Michaelis–Menten plot for determination of K_m (b) Kinetic analysis for determination of k_{cat}.

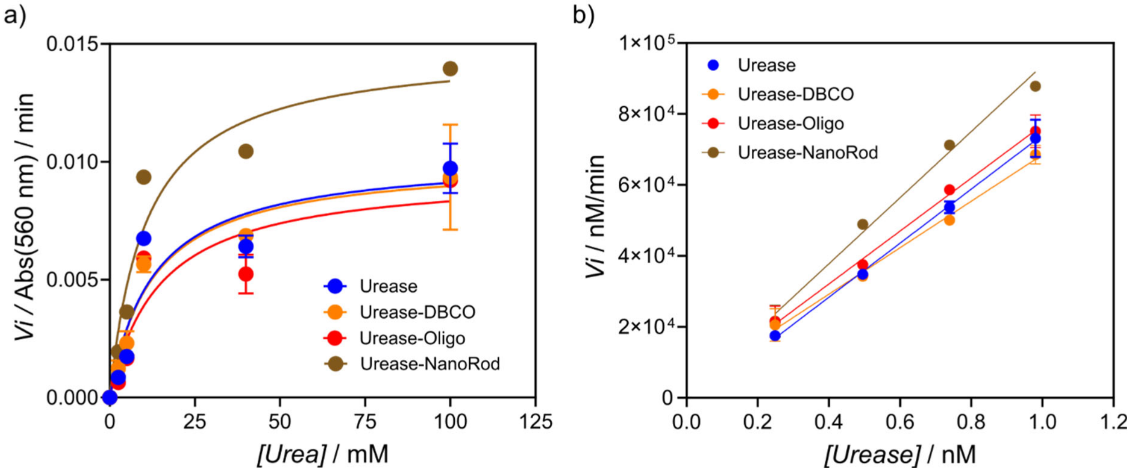

Figure S6 | Phoretic mobility b of NRs determined by first fitting micromotor speeds to experimental MSDs, and then using Eq. S1 with the term $\langle \nabla_{||} u \rangle$ obtained from BEM simulations. Since the micromotor speed is needed to determine phoretic mobilities, they could only be acquired for NR conditions that showed a net speed. The phoretic mobility is plotted against the degree of functionalization χ_0 and the degree of asymmetry φ.

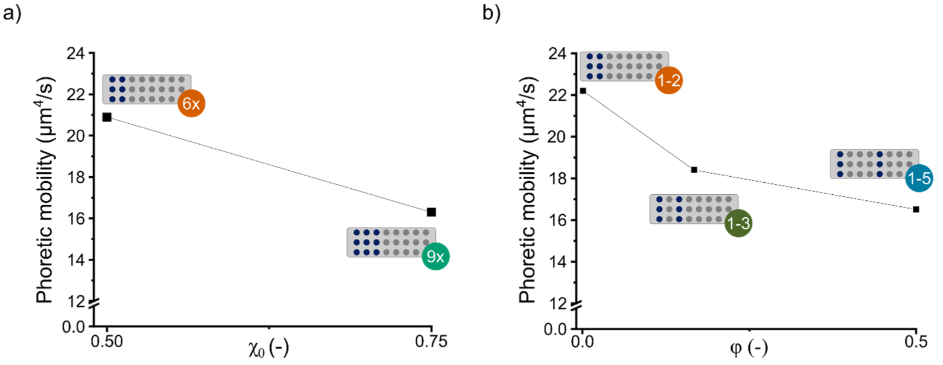

Figure S7 | Graphical representation of the metrics used for determining the a) degree of symmetry (φ) and b) the effective degree of asymmetry.

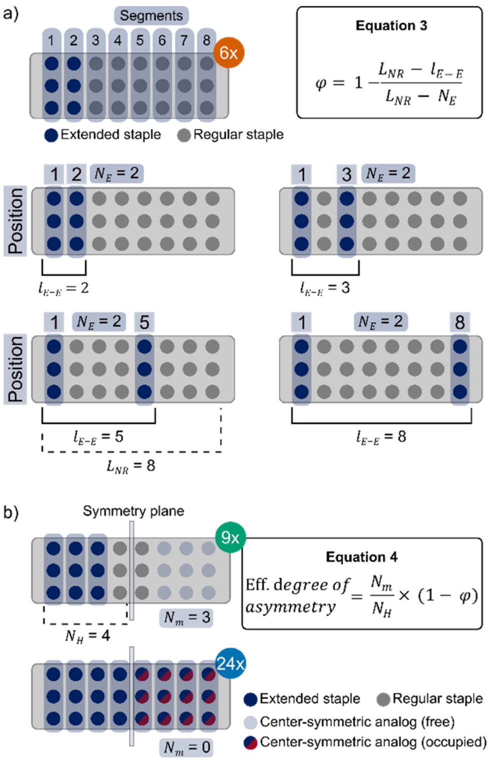

Figure S8 | Log-Log plots of the MSD profiles shown in Fig. 2a from which the global MSD exponent (α) was determined after performing a logarithmic fitting.

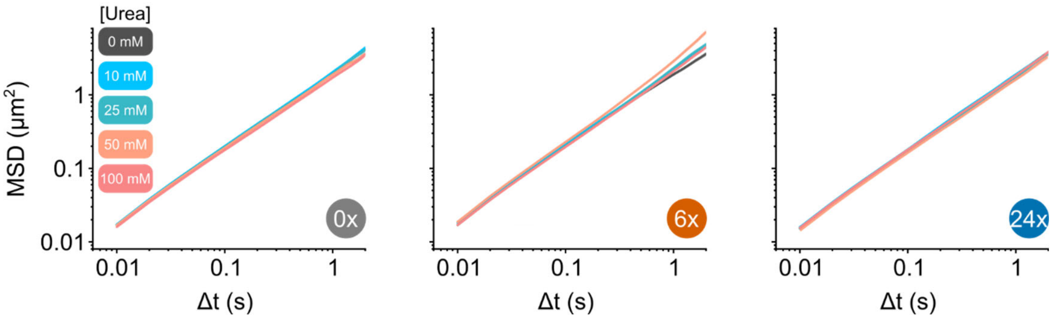

Table S1 | Handle-extended staple strands for urease immobilization on the 18-helix bundle DNA nanorod.

To assemble DNA nanorods for urease-oligo conjugation, appropriate unmodified staples were replaced with handle-extended staple strands. Bold nucleotides refer to the extended handle sequence. Labels: M, motor; F, fluorophore.

ID	Unmodified Staple ID	Anti-handle	Sequences (5' to 3')
mF2	Oligo 9	F	ACT GAC TGA CTG ACT GAC TG TTATCAATATTTAATTCGAGCTTCCCCTCA
mF3	Oligo 12	F	ACT GAC TGA CTG ACT GAC TG TTAGATTCACAACAGGTCAGGATAGCGTCC
mF4	Oligo 15	F	ACT GAC TGA CTG ACT GAC TG TTCTCATATATAAGAGGTCATTTAGTTTTG
mF5	Oligo 18	F	ACT GAC TGA CTG ACT GAC TG TTAGAAGCCAATATAATGCTGTAACGACGA
mF6	Oligo 21	F	ACT GAC TGA CTG ACT GAC TG TTGGTTGTAAGTACGGTGTCTGGAGGCATA
mF7	Oligo 24	F	ACT GAC TGA CTG ACT GAC TG TTAGCAAAACCAATTCTGCGAACACATTCA
mM1.1	Oligo 5	M	TCG TTC GAT CTT ATA TTC ACA TTTTTTATGCGCAAGAGTCTGGAGCAATAATGCC
mM1.2	Oligo 7	M	TCG TTC GAT CTT ATA TTC ACA TTTTTTAAAATCATGCTCCAAAGCGCGAAACAAACGCCACC
mM1.3	Oligo 123	M	TCG TTC GAT CTT ATA TTC ACA TTTTTTTTTCATCTTTCCCTTAGAATCCATAAATCAATATAAGCCAGC
mM2.1	Oligo 8	M	TCG TTC GAT CTT ATA TTC ACA TTTTTTGGAGAAGAAACTAGCATGTCAAATCACC
mM2.2	Oligo 10	M	TCG TTC GAT CTT ATA TTC ACA TTTTTTAATGCTTCATAAGGAATACACTAAAACATTTCAGG
mM2.3	Oligo 125	M	TCG TTC GAT CTT ATA TTC ACA TTTTTTACCAAGTAGACGCTGAGAAGATTAACAATTTCATTCCTCAAA
mM3.1	Oligo 11	M	TCG TTC GAT CTT ATA TTC ACA TTTTTTGTCACGAAAAGCCCCAAAAACTAGGTAA
mM3.2	Oligo 13	M	TCG TTC GAT CTT ATA TTC ACA TTTTTTAATACTGAACGGTGTGCCACTACGAAGGACTGAGT
mM3.3	Oligo 127	M	TCG TTC GAT CTT ATA TTC ACA TTTTTTTAATCGGGTCTGAGAGACTACAAGATGATGAAACAAATCAAC
mM4.1	Oligo 14	M	TCG TTC GAT CTT ATA TTC ACA TTTTTTTGCTGCATTGTAAACGTTAATTAGAACC
mM4.2	Oligo 16	M	TCG TTC GAT CTT ATA TTC ACA TTTTTTCCAGAGGAAGAGTATTTTTCATGAGGAATCCACAG
mM4.3	Oligo 129	M	TCG TTC GAT CTT ATA TTC ACA TTTTTTTAATATCATATAACTATATGTCGCGCAGAGGCGAACTTTAGG
mM5.1	Oligo 17	M	TCG TTC GAT CTT ATA TTC ACA TTTTTTGCGGGCCGTTAAATCAGCTCATTGCGGG
mM5.2	Oligo 19	M	TCG TTC GAT CTT ATA TTC ACA TTTTTTTAAAAACAACGTAAACGAGGGTAGCAACTGTCGTC
mM5.3	Oligo 131	M	TCG TTC GAT CTT ATA TTC ACA TTTTTTGCGCCTGCAAAGAACGCGAGATAACGGATTCGCCTATAATAC
mM6.1	Oligo 20	M	TCG TTC GAT CTT ATA TTC ACA TTTTTTTTCGCCAAATAATTCGCGTCTCTAAATC
mM6.2	Oligo 22	M	TCG TTC GAT CTT ATA TTC ACA TTTTTTGTAAGAGACGAGAATTTGCGGGATCGTCATTTTGC
mM6.3	Oligo 133	M	TCG TTC GAT CTT ATA TTC ACA TTTTTTCCAGACGTTCATCTTCTGACCATGAATATACAGTAATTCGAC
mM7.1	Oligo 23	M	TCG TTC GAT CTT ATA TTC ACA TTTTTTTTCCGGCATTAAATGTGAGCGAAGAATT
mM7.2	Oligo 25	M	TCG TTC GAT CTT ATA TTC ACA TTTTTTACTAATGTTTAATTATATATTCGGTCGCAGAAAGG
mM7.3	Oligo 135	M	TCG TTC GAT CTT ATA TTC ACA TTTTTTTAAGAGATGTGATAAATAAGGCAGAAATAAAGAAATTTTAAA
mM8.1	Oligo 26	M	TCG TTC GAT CTT ATA TTC ACA TTTTTTGACGACAAACAAACGGCGGATTAGTAGT
mM8.2	Oligo 28	M	TCG TTC GAT CTT ATA TTC ACA TTTTTTGTAGAAATTAAGAAGTTGCGCCGACAATCGTTGAA
mM8.3	Oligo 137	M	TCG TTC GAT CTT ATA TTC ACA TTTTTTCGCCAACTAATTACTAGAAAAAAGGGTTAGAACCTAACCACC

Table S2 | Sequences of unmodified staple strands of the 18-helix bundle DNA nanorod (corresponding to the handle-extended strands)

Locations of the 5’ and 3’ end are indicated using the reference helix number, with the reference nucleotide position denoted in brackets.

Staple ID	Location of 5' end	Location of 3' end	Sequence
F2	4[139]	6[140]	ATCAATATTTAATTCGAGCTTCCCCTCA
F3	4[181]	6[182]	AGATTCACAACAGGTCAGGATAGCGTCC
F4	4[223]	6[224]	CTCATATATAAGAGGTCATTTAGTTTTG
F5	4[265]	6[266]	AGAAGCCAATATAATGCTGTAACGACGA
F6	4[307]	6[308]	GGTTGTAAGTACGGTGTCTGGAGGCATA
F7	4[349]	6[350]	AGCAAAACCAATTCTGCGAACACATTCA
M1.1	2[97]	4[98]	ATGCGCAAGAGTCTGGAGCAATAATGCC
M1.2	6[97]	9[90]	AAAATCATGCTCCAAAGCGCGAAACAAACGCCACC
M1.3	14[83]	17[83]	TTTCATCTTTCCCTTAGAATCCATAAATCAATATAAGCCAGC
M2.1	2[139]	4[140]	GGAGAAGAAACTAGCATGTCAAATCACC
M2.2	6[139]	9[132]	AATGCTTCATAAGGAATACACTAAAACATTTCAGG
M2.3	14[125]	17[125]	ACCAAGTAGACGCTGAGAAGATTAACAATTTCATTCCTCAAA
M3.1	2[181]	4[182]	GTCACGAAAAGCCCCAAAAACTAGGTAA
M3.2	6[181]	9[174]	AATACTGAACGGTGTGCCACTACGAAGGACTGAGT
M3.3	14[167]	17[167]	TAATCGGGTCTGAGAGACTACAAGATGATGAAACAAATCAAC
M4.1	2[223]	4[224]	TGCTGCATTGTAAACGTTAATTAGAACC
M4.2	6[223]	9[216]	CCAGAGGAAGAGTATTTTTCATGAGGAATCCACAG
M4.3	14[209]	17[209]	TAATATCATATAACTATATGTCGCGCAGAGGCGAACTTTAGG
M5.1	2[265]	4[266]	GCGGGCCGTTAAATCAGCTCATTGCGGG
M5.2	6[265]	9[258]	TAAAAACAACGTAAACGAGGGTAGCAACTGTCGTC
M5.3	14[251]	17[251]	GCGCCTGCAAAGAACGCGAGATAACGGATTCGCCTATAATAC
M6.1	2[307]	4[308]	TTCGCCAAATAATTCGCGTCTCTAAATC
M6.2	6[307]	9[300]	GTAAGAGACGAGAATTTGCGGGATCGTCATTTTGC
M6.3	14[293]	17[293]	CCAGACGTTCATCTTCTGACCATGAATATACAGTAATTCGAC
M7.1	2[349]	4[350]	TTCCGGCATTAAATGTGAGCGAAGAATT
M7.2	6[349]	9[342]	ACTAATGTTTAATTATATATTCGGTCGCAGAAAGG
M7.3	14[335]	17[335]	TAAGAGATGTGATAAATAAGGCAGAAATAAAGAAATTTTAAA
M8.1	2[391]	4[392]	GACGACAAACAAACGGCGGATTAGTAGT
M8.2	6[391]	9[384]	GTAGAAATTAAGAAGTTGCGCCGACAATCGTTGAA
M8.3	14[377]	17[377]	CGCCAACTAATTACTAGAAAAAAGGGTTAGAACCTAACCACC

Table S3 | Anti-handle for urease labeling. This 3’ azide-oligo was used for NHS coupling to the protein of interest. Molecular weight of the ssDNA strand is 6561.69 g/mol (ssDNA: Mass to Moles Convertor; NEBioCalculator).

ID	Sequence (5’ to 3’)
M(otor)	TGT GAA TAT AAG ATC GAA CGA - N3

Table S4 | Fluorescently labeled imagers

To label DNA nanostructures, complementary imager (F) strands were used during the self-assembly of the DNA nanostructure.

ID	Sequences (5’ to 3’)
F(luorophore)	CAG TCA GTC AGT CAG TCA GT - ATTO647N

Table S5 | Sequences of unmodified staple strands of the 18-helix bundle DNA nanorod.

Locations of the 5’ and 3’ end are indicated using the reference helix number, with the reference nucleotide position denoted in brackets.

Staple ID	Location of 5' end	Location of 3' end	Sequence
1	0[51]	14[42]	AAGACACCGCCTAACTGGCGCGGTAAGCCAACAGAGAT
2	0[93]	14[94]	GGAGAAAAATAACAGTACTTGAAACAAG
3	0[114]	17[104]	GGTTGCTGAATGAATTACCTTTTTAATGGACTAAAGC
4	0[135]	14[136]	CAACCCTCAAATTACATGTCAATAAGAA
5	0[156]	17[146]	TCGGTTGGCAAACATCAAGAAAACAAAATTATCAATAT
6	0[177]	14[178]	TGTAGGAATTGCAAAAGCTTTTTATAGA
7	0[198]	17[188]	TGCAAAATATTTATTCATTTCAATTACCTGAGAGGAAG
8	0[219]	14[220]	AGCACAACTAACAAAATAAATGCTCTGA
9	0[240]	17[230]	GCCTCAATAGGATTGCTTTGAATACCAAGTTATAGATT
10	0[261]	14[262]	AACGATTTAGGAAACAAAAACTTTAGCT
11	0[282]	17[272]	CTTACAAACAACAGTACCTTTTACATCGGGAAAGTATT
12	0[303]	14[304]	TTGATTAAATACGTCAGTAAATTTTAAA
13	0[324]	17[314]	CGGTTATTAATTGCGTAGATTTTCAGGTTTACCTTTGC
14	0[345]	14[346]	ATTGTAACATCGTAAAACGTTAAATTTC
15	0[366]	17[356]	GAAACAAAGAACCATATCAAAATTATTTGCATATCATT
16	0[387]	14[388]	CACGCGGAATATAATGGAGCCTGTGCCA
17	0[408]	17[398]	TCATGATTATTTGTTTGGATTATACTTCTGATATCATC
18	0[419]	3[412]	AGTGAGCCATACGAAACCGTGCATCTGCAATGGGA
19	1[73]	17[58]	TCAATTAAAGACGCTGAGTGTGAGTGAATAACCTTGCTTCTG
20	2[34]	4[35]	CCGATTTGAGAAAGGAAGGGAGCGCGTA
21	2[55]	2[56]	TCGGAACGAAAGGAGCGGGCGCTAGGAATGTAAAGCACTAAA
22	2[76]	4[77]	TTAGTGCAGGCTATCAGGTCATTTTTGA
23	2[118]	4[119]	CTTCTAAAATCGATGAACGGTCAACCGT
24	2[160]	4[161]	GGCCAGTGTACCCCGGTTGATGAGAAAG
25	2[202]	4[203]	GGTAACGTTGTATAAGCAAATATGCAAT
26	2[244]	4[245]	CCAGCTGTAAAATTCGCATTACAACGCA
27	2[286]	4[287]	CTGTTGGACCAATAGGAACGCCATTATG
28	2[328]	4[329]	CGGAAACCTGTAGCCAGCTTTATAAAGC
29	2[370]	4[371]	AAGATCGACCCGTCGGATTCTATAAATC
30	3[413]	6[413]	TAGGTCAAGGTGGCATCAATTCTGTTTAGCTATATACGAACT
31	4[34]	7[34]	ACCACCAGCGTACTATGGTTGAAACAGGAGGCCGAGAATCCT
32	4[55]	4[56]	GTAGCGGGAGCACGTATAACGTGCTTTCACGCGCTGGCAAGT
33	4[76]	6[77]	GAGATCTAGAAGCAAAGCGGAACCCTGA
34	4[107]	0[115]	AATACAAGAGGTGGTTGCCCGCTTCTAATCTATAG
35	4[118]	6[119]	TCTAGCTGAAAGACTTCAAATTTCAGAA
36	4[149]	0[157]	TCAATCATATGCCAAGCGATACCGACAGTGCGAAA
37	4[160]	6[161]	GCCGGAGAACCAGACCGGAAGGTCATAA
38	4[191]	0[199]	GTGAGGAAGACCAGGGTGTGGGCACGAATATGGTT
39	4[202]	6[203]	GCCTGAGTACCTTTAATTGCTAGTAAAA
40	4[233]	0[241]	TTTATTTTGTGCGAAAGACATAAATCATTTCCACC
41	4[244]	6[245]	AGGATAATGGCTTAGAGCTTAGCGAGAG
42	4[275]	0[283]	CTTTTTTTTAGAAGGGCATGAGTAAACAGGGTTTT
43	4[286]	6[287]	ACCCTGTATGTTTTAAATATGATCATAA
44	4[317]	0[325]	AAGGGCCTTCCAGGCAAATAAAGACGGAGGACGCG
45	4[328]	6[329]	CTCAGAGATTCCATATAACAGATAACGC
46	4[359]	0[367]	GCAAGTAACACACTCCATCATGGTCATAGCTTCGG
47	4[370]	6[371]	ATACAGGTTTAGTTTGACCATCAGTTGA
48	4[401]	0[409]	TAATGACCGTCAGTTTGACAATTCCACACAATAAC
49	5[46]	0[52]	GACTCACGCTAGAAAGCCCTAAAGTCAAGTTTTTTGGGGTCA
50	5[88]	0[94]	ATCGTAGCTATTGCCTGCGACTTAACAATGTCCCGCCAGTTT
51	5[130]	0[136]	CGTTGATATTAATCGTACCAGGGTCTCGCCCTGGAGTGAAAT
52	5[172]	0[178]	CTCAAAGGGTAATCAGACGTTGTACCATCTGTAAGCAAATCC
53	5[214]	0[220]	TTGATTTTAAATTTAAAAGGCGATTTGAATCGGCTGACTTGC
54	5[256]	0[262]	CTGTTTATTTAATTTTTTCTTCGCTCTGACCTCCTGGTGGGC

55	5[298]	0[304]	TAACCAAAAACATCAAATTCAGGCTACGTGGTGCTTGTCGTA
56	5[340]	0[346]	TTCTTAAGCACATCAACACCGCTTGTACCGAGCTCGAACTGC
57	5[382]	0[388]	TACACATCCACCGTGGGGTATCGGGTGTGAAATTGTTACGCT
58	6[55]	6[56]	TCAGAGCTAATCAGTGAGGGCCTGATAAAGTCCTCGTTAGAA
59	6[76]	9[69]	CTATTATATTGTGTGGAGATTTGTATCATAGTACC
60	6[118]	9[111]	AACGAGAAACGAGGTTGACCCCCAGCGACCTCAGA
61	6[160]	9[153]	ATATTCAAACTTTGCTAAAACGAAAGAGTAGGAAC
62	6[202]	9[195]	TGTTTAGATAGGCTTTAAACGGGTAAAAAAACTAC
63	6[244]	9[237]	GCTTTTGCGGATATGAGGCTTTGAGGACTAGCGTA
64	6[286]	9[279]	CCCTCGTCAGTGAAGCAGCGAAAGACAGTAAATGA
65	6[328]	9[321]	CAAAAGGAGTAAATTGCAGGGAGTTAAACAGTTTC
66	6[370]	9[363]	GATTTAGTGTGAATACCATCGCCCACGCTTGCGAA
67	6[412]	9[405]	AACGGAACAGTCAGTTAAACAGCTTGATAGGCTCC
68	9[49]	9[48]	GCCTGAGTAGAATTTCCCACCGAGTAAAAGAGTCTATCACTT
69	9[70]	12[70]	GCCACCCCACCGTACTCAGGATTAAAGGTGAATTAATTGACG
70	9[91]	11[90]	CTCAGAATATAGCCCGGAATACACCGAC
71	9[102]	4[108]	CACTTATACCTGTTACTTAGCCGGATGACCAGAAGCCCGATA
72	9[112]	12[112]	GCCACCACGTCGAGAGGGTTGAATTAGAGCCAGCACAAAAGG
73	9[133]	11[132]	GATAGCACAGTACCAGGCGGACAGTAGC
74	9[144]	4[150]	CAAGCAAAAGGAACCGAACTGACCTTGAATCCAAAGCGACAG
75	9[154]	12[154]	CCATGTATAGGATTAGCGGGGCAAGGCCGGAAACGCACAATC
76	9[175]	11[174]	TTCGTCAGAGACTCCTCAAGATGAAACC
77	9[186]	4[192]	TACTACGTAATACAGACCAGGCGCACTGGATTAGAGAGTAAT
78	9[196]	12[196]	AACGCCTACATGAAAGTATTACGTAATCAGTAGCGATAAAAG
79	9[217]	11[216]	ACAGCCCTTCGGAACCTATTACAAGTTT
80	9[228]	4[234]	AGTTAAAGACATCTTGACAAGAACCAAAAGATTGCGGAAAAT
81	9[238]	12[238]	ACGATCTCAGTTAATGCCCCCACTGTAGCGCGTTTTGTTAGC
82	9[259]	11[258]	TTTCCAGGAGTAACAGTGCCCCATTTTC
83	9[270]	4[276]	TAGCATCGGACAAAGCTGCTCATTTTACCAGGCTCAACAATA
84	9[280]	12[280]	ATTTTCTTTTTAACGGGGTCATATTAGCGTTTGCCCCCAAAA
85	9[301]	11[300]	TAAACAACAGGAGTGTACTGGCATAATC
86	9[312]	4[318]	CAAGGCCGCTACACCAGAACGAGTAATTACGAAGTTTCCATA
87	9[322]	12[322]	AGCGGAGCATACATGGCTTTTCAGAGCCACCACCGGTTACCA
88	9[343]	11[342]	AACAACTATTTACCGTTCCAGCTCCCTC
89	9[354]	4[360]	GAAATAACCGTCAACTTTAATCATGAATACCGAGTAGACAAG
90	9[364]	12[364]	TAATAATAATGGAAAGCGCAGCAGAACCGCCACCCCCGAAGC
91	9[385]	11[384]	AATCTCCATAAATCCTCATTACACCACC
92	9[396]	4[402]	AAAACCGATACTGGCTCATTATACCAACATTCAATAACCTAC
93	9[406]	12[406]	AAAAGGAGGCCTTGATATTCACAGAACCACCACCAATAATAA
94	10[37]	5[45]	GGTTAATAACGTCCATCGAGAAGTGTTTTTAGGGAGCTCTTT
95	10[79]	5[87]	TATTCAGAACGTACAACCGAAATCCGCGACCGGTCTTTTTGC
96	10[121]	5[129]	TGCCCCTCATCTCATCTCGCAGACGGTCAATTAAACAGATCG
97	10[163]	5[171]	GATCCGTAACCACCAACAAAGAGGACAGATGCGGAATCCAAA
98	10[205]	5[213]	GAAGTAGCATGTTTCCAGGCTGACCTTCATCGGGTAATCCTT
99	10[247]	5[255]	AAAAAAGTTTGGCTACATCATTACCCAAATCCAAAATAATTG
100	10[289]	5[297]	AAGGTATGGGACCCTCATAAGGCTTGCCCTGCAACACTCAAC
101	10[331]	5[339]	CGTTGAGAATTGAGGCTTGGGCTTGAGATGGCAGATACTTGA
102	10[373]	5[381]	CAGTTTTTCAGACAACATACCTTATGCGATTGATTCATTAGA
103	11[49]	11[48]	GAAATACCTACACAGGGAACTCAAACTATCGGCCTGCTCATG
104	11[91]	14[84]	TTGAGCCATTGAGGGAGGGAACCGGTATTTTTTAT
105	11[133]	14[126]	ACCATTAATGGTTTACCAGCGCTTGCGGGTATTAA
106	11[175]	14[168]	ATCGATACACGGAATAAGTTTTTTTGCACAATCAA
107	11[217]	14[210]	GCCTTTACATACATAAAGGTGTAACGAGAGAAAAA
108	11[259]	14[252]	GGTCATAAAGACTCCTTATTAAAACAGCGCAGAAC
109	11[301]	14[294]	AAAATCAAAACGCAATAATAATTTTGTTATTCTGT
110	11[343]	14[336]	AGAGCCGGTAAGCAGATAGCCAGAATAACCAGTAA
111	11[385]	14[378]	CTCAGAGGAAATAGCAATAGCAACTGAATTAACAA
112	12[69]	15[73]	GAAATTAAATCAGATCATTACCGCGCCCAAATCGTCGCTATTAATT
113	12[111]	15[115]	GCGACATGCGTTTTTCATCGAGAACAAGACATAGCGATAGCTTAGA
114	12[153]	15[157]	AATAGAATTAAATCTCCTTATCATTCCAGTGAATTTATCAAAATCA

115	12[195]	15[199]	AAACGCATATCCTGAATTTACGAGCATGACCTCCGGCTTAGGTTGG
116	12[237]	15[241]	AAACGTACTAATTTACAATAGATAAGTCGATGCAAATCCAATCGCA
117	12[279]	15[283]	GAACTGGCAATCCATAAACAACATGTTCTTCAAATATATTTTAGTT
118	12[321]	15[325]	GAAGGAAAAAAATAGGTACCGACAAAAGGAATGGTTTGAAATACCGA
119	12[363]	15[367]	CCTTTTTAGCGCATTTAGGCAGAGGCATTAAGAATAAACACCGGAA
120	12[405]	15[409]	GAGCAAGTCAGAGGCTTAATTGAGAATCTTAGTATCATATGCGTTA
121	13[49]	13[48]	TTCTGTAGCAAGCATTTTTTGACGCTCAATCGTCTAGGGACA
122	14[41]	17[41]	AGAACCCTATTAGTCTTTAATATAGCCCTAAAACAGGCGGTC
123	14[83]	17[83]	TTTCATCTTTCCCTTAGAATCCATAAATCAATATAAGCCAGC
124	14[93]	9[101]	CCGTCTAAGAACGCGAGTCAACCGATTTGGGATATAAGCCGC
125	14[125]	17[125]	ACCAAGTAGACGCTGAGAAGATTAACAATTTCATTCCTCAAA
126	14[135]	9[143]	CGGGAGGTTTTGAAGCCAATTCATCCATTAGTTTTGCTAGCC
127	14[167]	17[167]	TAATCGGGTCTGAGAGACTACAAGATGATGAAACAAATCAAC
128	14[177]	9[185]	AACCCCAGCTACAATTTAAGACACGCAGCACAGAGGCTCCAG
129	14[209]	17[209]	TAATATCATATAACTATATGTCGCGCAGAGGCGAACTTTAGG
130	14[219]	9[227]	ACACGTCTTTCCAGAGCGAAAATAGCGTCAGTGCCTATTCAT
131	14[251]	17[251]	GCGCCTGCAAAGAACGCGAGATAACGGATTCGCCTATAATAC
132	14[261]	9[269]	AATCATATTATTTATCCCATGATTGCCCCCTGTGCCTTACGT
133	14[293]	17[293]	CCAGACGTTCATCTTCTGACCATGAATATACAGTAATTCGAC
134	14[303]	9[311]	GTATAACGTCAAAAATGACCGAGGCCGGAACGATGATACTTT
135	14[335]	17[335]	TAAGAGATGTGATAAATAAGGCAGAAATAAAGAAATTTTAAA
136	14[345]	9[353]	GAGCATAAAAACAGGGAAAGAAAACCACCCTTCTCTGAAAAG
137	14[377]	17[377]	CGCCAACTAATTACTAGAAAAAAGGGTTAGAACCTAACCACC
138	14[387]	9[395]	TATCACCCTGAACAAAGAAACAATCCGCCACCAAACAAAAAA
139	15[32]	10[38]	GGCTTCTGACCGACCAGTAATAAAGAAATGGGAAAAACTGCT
140	15[74]	10[80]	AATGTAGGAATATAGAAGGCTTATGGTAAATTCACCGTGGTG
141	15[116]	10[122]	TTAACCGCACAGCGAACCTCCCGACCAAAGAAAATCACTAAG
142	15[158]	10[164]	TAGCTGTCTTAAGATTAGTTGCTAATTTTGTTCACCAAGAAG
143	15[200]	10[206]	GTTCCATCCTAATCTTACCAACGCGCAACATACAGAATTTCT
144	15[242]	10[248]	AGATTTATCAGCCAGTTACAAAATCGCAGTATCATCGGGTAT
145	15[284]	10[290]	AATACGACAAAATAAGAAACGATTCGGAATAATCTTTTTAAT
146	15[326]	10[332]	CCGATATAAACAGCCTTTACAGAGGAACAAAGAACCGCTAAG
147	15[368]	10[374]	TCAATGTAATTAGACGGGAGAATTTATCTTATCAGAGCAAGC
148	15[410]	10[416]	TACAGTAGGGGTAATTGAGCGCTAAAGCCCAGAGCCGCGACG
149	17[42]	2[35]	AGTATTAGGCGAAAAACCGTCACCCAAAGGAGCCC
150	17[59]	1[72]	CAACAGTGCCAACGTGGACTCCAACGTCGAGGTGCCGAATTG
151	17[84]	2[77]	AGCAAATACAAGAGTCCACTACCTTATGAGTGTCC
152	17[126]	2[119]	TATCAAAAAGAATAGCCCGAGATTTACGGGATGTT
153	17[168]	2[161]	AGTTGAATTGATGGTGGTTCCGGCCCTGAAACGAC
154	17[189]	2[182]	GTTATCTCCCAGCAGGCGAAACTCGTCGTTTCCCA
155	17[210]	2[203]	AGCACTAAAGCGGTCCACGCTAGGGGCCTAAGTTG
156	17[252]	2[245]	ATTTGAGAGCTGATTGCCCTTTCCGAACTATTACG
157	17[273]	2[266]	AGACTTTTTCACCAGTGAGACTGGTGTAGATCGGT
158	17[294]	2[287]	AACTCGTGGCGCCAGGGTGGTCTTAAGCTGCGCAA
159	17[315]	2[308]	CCGAACGGGAGAGGCGGTTTGTACCTCGAGCGCCA
160	17[336]	2[329]	AGTTTGAAATGAATCGGCCAATCCCCGGCTGGTGC
161	17[357]	2[350]	TTGCGGAACCTGTCGTGCCAGTTCGTAAGCCAGCT
162	17[378]	2[371]	AGAAGGATGCCCGCTTTCCAGGTTTCCTCCTCAGG
163	1[15]	0[15]	AAAACACTACGTGAACCATCTATCAGGGCGATGGCCAAAA
164	3[8]	2[8]	AAAAAGCCGGCGAACGTGGCAGAGCTTGACGGGGAAAAAA
165	5[8]	4[8]	AAAATGCGCCGCTACAGGGCCACCCGCCGCGCTTAAAAAA
166	7[8]	6[8]	AAAACAGGAACGGTACGCCATTAAAGGGATTTTAGAAAAA
167	8[34]	8[12]	ACGCAAATTAACCGTTGTAAAAA
168	9[12]	9[34]	AAAAGCAATACTTCTTTGATTAG
169	10[34]	10[12]	AATATCCAGAACAATATTAAAAA
170	11[12]	11[34]	AAAACCGCCAGCCATTGCAACAG
171	12[34]	12[12]	ATTATTTACATTGGCAGATAAAA
172	13[12]	14[5]	AAAATCACCAGTCACACTGAAAGCGTAAGAATACGAAAA
173	15[5]	15[31]	AAAATGGCACAGACAATATTTTTGAAT
174	17[5]	17[27]	AAAAACCAGCAGAAGATAAAACA

175	17[28]	16[5]	GAGGTGATCGCCATTAAAAATACCGAACGAACCAAAA
176	0[442]	17[432]	AAAAAAGCCTGGGGTGTCAAAA
177	2[435]	1[442]	AAAATGGGCGCATCGTGCCGGAAGCATAAAGTGTAAAAA
178	4[435]	3[435]	AAAACGCGAGCTGAAACGTTGGTGTAGAAAAA
179	6[435]	5[435]	AAAATACGTTAATAAATTTCATTTGGGGAAAA
180	8[439]	9[439]	AAAAAGCTTGCTTTCGAGGTATTGTATCGGTTTATCAAAA
181	10[415]	7[435]	ATTGCCTTTAGAATTTCGACGTTGGGAAGAAAAATCAAAA
182	10[440]	11[440]	AAAAAGGTTGAGGCAGGTCACGCCAGCATTGACAGGAAAA
183	12[439]	13[439]	AAAACCACAAGAATTGAGTTATATCAGAGAGATAACAAAA
184	14[432]	15[432]	AAAAAAAGCCAACGCTCAACAAATTCTTACCAGTATAAAA
185	16[432]	0[420]	AAAAATCAATATAATCCTGACAGATGATGGCAATCCTAATG
186	2[97]	4[98]	ATGCGCAAGAGTCTGGAGCAATAATGCC
187	4[97]	6[98]	GGAGAGGAAAAAGATTAAGAGTAAATCA
188	6[97]	9[90]	AAAATCATGCTCCAAAGCGCGAAACAAACGCCACC
189	17[105]	2[98]	ATCACCTTGAGTGTTGTTCCAAAATAACTGAATTC
190	2[139]	4[140]	GGAGAAGAAACTAGCATGTCAAATCACC
191	4[139]	6[140]	ATCAATATTTAATTCGAGCTTCCCCTCA
192	6[139]	9[132]	AATGCTTCATAAGGAATACACTAAAACATTTCAGG
193	17[147]	2[140]	CTGGTCAGCAAAATCCCTTATACTCTATTTTCTCA
194	2[181]	4[182]	GTCACGAAAAGCCCCAAAAACTAGGTAA
195	4[181]	6[182]	AGATTCACAACAGGTCAGGATAGCGTCC
196	6[181]	9[174]	AATACTGAACGGTGTGCCACTACGAAGGACTGAGT
197	2[223]	4[224]	TGCTGCATTGTAAACGTTAATTAGAACC
198	4[223]	6[224]	CTCATATATAAGAGGTCATTTAGTTTTG
199	6[223]	9[216]	CCAGAGGAAGAGTATTTTTCATGAGGAATCCACAG
200	17[231]	2[224]	AGAGCCGTGGCCCTGAGAGAGGCATTTCGGGGATG
201	2[265]	4[266]	GCGGGCCGTTAAATCAGCTCATTGCGGG
202	4[265]	6[266]	AGAAGCCAATATAATGCTGTAACGACGA
203	6[265]	9[258]	TAAAAACAACGTAAACGAGGGTAGCAACTGTCGTC
204	2[307]	4[308]	TTCGCCAAATAATTCGCGTCTCTAAATC
205	4[307]	6[308]	GGTTGTAAGTACGGTGTCTGGAGGCATA
206	6[307]	9[300]	GTAAGAGACGAGAATTTGCGGGATCGTCATTTTGC
207	2[349]	4[350]	TTCCGGCATTAAATGTGAGCGAAGAATT
208	4[349]	6[350]	AGCAAAACCAATTCTGCGAACACATTCA
209	6[349]	9[342]	ACTAATGTTTAATTATATATTCGGTCGCAGAAAGG
210	2[391]	4[392]	GACGACAAACAAACGGCGGATTAGTAGT
211	4[391]	6[392]	AGCATTAATTTCGCAAATGGTATTACAG
212	6[391]	9[384]	GTAGAAATTAAGAAGTTGCGCCGACAATCGTTGAA
213	17[399]	2[392]	ATATTCCCATTAATTGCGTTGTCCGCTCAGGGGAC

Table S6 | Kinetic parameters illustrating the urease activity of the different stages in the functionalization process.

Sample	$^{(app)}K_m$ (mM)	$^{(app)}kcat$ (min^{-1}, $x10^4$)	C.E. (min^{-1} mM^{-1}, $x10^3$)	Rel. C.E. (%)
Urease	12.63 ± 4.99	7.6 ± 0.3	6.0	100
Urease-DBCO	12.52 ± 3.70	6.5 ± 0.3	5.2	87
Urease-Oligo	14.52 ± 6.34	7.5 ± 0.4	5.1	85
Urease-NR	10.68 ± 2.59	9.3 ± 0.5	8.7	145

Table S7 | Parameters used for estimation of Damköhler number

R_{NR} (nm)	100 (over-estimation)
d_{urea} (m^2/s)	1.30×10^9 (water)
d_{urea} (m^2/s)	1.4×10^8 (60% (v/v) glycerol)
c_{sub} (mM)	50
c_{sub} (molecules/m^3)	3.022×10^{-2}
k_{cat} (min^{-1})	9.3×10^4
Da (-)	1.8×10^{-5} (water)
Da (-)	1.7×10^{-4} (60% (v/v) glycerol)

This method is based on the determination of the Damköhler number by Song et al.[2]

The relative rate of the enzymatic reaction versus substrate diffusion can be estimated by the Damköhler number:

$$Da = \frac{rR}{dc_{sub}}$$

where r is the reaction rate, R is the hydrodynamic radius of the NR (over-estimation), d is the diffusioncoefficient of urea and c_{sub} is the concentration of urea far from the NR. The reaction rate r can be estimated as the product of the maximum turnover rate k_{cat} of urease multiplied by the surface density of the enzymes Γ_0.

$$r \approx k_{cat}\Gamma_0$$

We estimated surface density of the enzymes in the case of maximum packing (full coverage; 24x ureases. Since the inter-enzyme distance in that case is 14 nm, we assumed each urease to occupy the area of one third of a cylinder with a diameter (d) of 15 nm and height (h) of 14 nm.

$$\Gamma_0 = \frac{1}{\left(\frac{\pi dh}{3}\right)}$$

4. References

[1] M. N. Popescu, W. E. Uspal, S. Dietrich, “Self-diffusiophoresis of chemically active colloids” *The European Physical Journal Special Topics 2016 225:11* 2016, *225*, 2189–2206.

[2] S. Song, A. F. Mason, R. A. J. Post, M. De Corato, R. Mestre, N. A. Yewdall, S. Cao, R. W. van der Hofstad, S. Sanchez, L. K. E. A. Abdelmohsen, J. C. M. van Hest, “Engineering transient dynamics of artificial cells by stochastic distribution of enzymes” *Nat Commun* 2021, *12*, 1–9.

Model and Simulation Section

1 Problem statement and solution approach

Our programmable motors self-propel by converting fuel into product. Since only part of the micromotor surface is catalytically active, there will be an asymmetric product particle concentration around the micromotor, which leads to propulsion through diffusiophoresis. Following the model for self-diffusiophoresis of active Brownian particles by Popescu et al. [3], the boundary value problem of interest is given as

$$\begin{cases} \nabla^2 u(\mathbf{r}) = 0 \quad \text{for } \mathbf{r} \in \mathbb{R}^2 \backslash C_P, \\ -\mathbf{n} \cdot \nabla u(\mathbf{r}) = \begin{cases} 0 & \text{no enzyme} \\ \frac{K}{D} & \text{enzyme} \end{cases} & \text{for } \mathbf{r} \in \partial C_P, \\ u(\mathbf{r}) = 0 \quad \text{for } |\mathbf{r}| \to \infty, \end{cases}$$

where u is the product particle concentration, $\mathbf{n}$ the normal vector to the surface (we use boldface to denote vectors and matrices, as opposed to scalar quantities), K the catalytic activity per unit surface, D the product particle diffusion coefficient, and C_P the micromotor surface. Once u (or merely its gradient tangent to the micromotor surface) is known, the slip velocity at the boundary of the micromotor (as a function of position at the boundary) is computed through

$$\mathbf{v}_{\text{slip}} = -b\nabla_{||} u,$$

after which the net propulsion velocity of the micromotor is computed as the negative average of the slip velocity over the surface:

$$\mathbf{v}_{\text{p}} = -\langle \mathbf{v}_{\text{slip}} \rangle.$$

Here, $\nabla_{||}$ is the gradient tangent to the micromotor surface C_P and b the phoretic mobility, which is assumed to be constant over the micromotor surface.

We aim to solve the boundary value problem using the Boundary Element Method (BEM). The aim of the BEM is to massage the PDE into an integral equation over just the boundary of the computational domain, so that a discretization of the entire domain is not needed. The BEM is especially suitable for this problem due to its infinitely large domain, which makes methods like the Finite Element Method and the Finite Volume Method less suitable [2]. Our boundary value problem is an exterior Laplace problem, since the

domain of interest lies outside the volume enclosed by the micromotor. However, we first develop a model for the simpler interior Laplace problem. Furthermore, we consider first a spherical micromotor, and a rod-shaped one only afterward. Note that our models are 2D, while experimental data of course comes from 3D, of which a 2D projection is extracted.

The remainder of this document is organised as follows. In Section 2, we present a (mostly) self-contained derivation of boundary integral equations for the 2D Laplace problem in several geometries. First, we consider the interior of a circular domain in Section 2.1, after which we consider the exterior of a circular domain in Section 2.2. In the latter, we continue the analysis by discretizing the boundary integral equations. In Section 2.3, we consider the exterior of a rectangular (rod-shaped) domain, where we include a full Python implementation of the discretized boundary integral equations for this geometry.

2 Boundary element method

We follow the book by Katsikadelis [2] in developing a boundary integral formulation of the boundary value problem, discretizing the resulting equation and solving it. We include extra details, drawing inspiration from the PhD thesis by Dijkstra [1].

We start with the interior Laplace problem.

2.1 Interior Laplace problem in 2D

The strong formulation of the (generalized) boundary value problem reads

$$\begin{cases} \nabla^2 u(x,y) = f(x,y) & \forall x, y \in \Omega \\ \alpha u(x,y) + \beta \frac{\partial u(x,y)}{\partial n} = \gamma & \text{on } \Gamma := \partial\Omega, \end{cases} \tag{1}$$

for an arbitrary function $f : \mathbb{R}^2 \to \mathbb{R}$, arbitrary constants $\alpha, \beta, \gamma \in \mathbb{R}$. Here, $\frac{\partial}{\partial n}$ denotes the partial derivative in the direction of the normal vector $\mathbf{n}$ to the surface. We first find the fundamental solution/freespace Green's function corresponding to this elliptic partial differential equation. Using this fundamental solution, we will derive the weak formulation, which leads to a boundary integral equation for u.

2.1.1 Fundamental solution

The fundamental solution is the response of the homogeneous part of the PDE to a unit point source, i.e.,

$$\nabla^2 v = \delta_P, \tag{2}$$

where δ_P is the Dirac delta function at a given point P. Note that it makes sense to determine this v, which denotes the fundamental solution, since the source term f can be decomposed into a sum of Dirac delta functions.

We consider a unit point source at a point $P = (x, y)$ in the xy-plane, so that it has density $\delta_P(Q)$ at any point Q. Then, we can use polar coordinates to convert (2) into

$$\frac{1}{r}\frac{\mathrm{d}}{\mathrm{d}r}\left(r\frac{\mathrm{d}v}{\mathrm{d}r}\right) = 0, \text{ for } r \neq 0, \tag{3}$$

where $r = |Q - P|$, since the Dirac delta vanishes everywhere except at $r = 0$. The other term in the Laplacian vanishes by axisymmetry of the fundamental solution. We get the additional condition that $\nabla^2 v(P) = \delta_P(P) = \infty$. We can integrate (3) twice to get

$$r\frac{\mathrm{d}v}{\mathrm{d}r} = A \implies v = A \ln r + B, \text{ for } A, B \in \mathbb{R}. \tag{4}$$

We are simply looking for a particular solution, so we set $B = 0$ for convenience. To determine A, we consider a small circular domain Ω_ε centered around P ($r = 0$) with radius $\varepsilon > 0$. We now integrate the Laplacian of v over this domain and use Green's second identity:

$$\int_{\Omega_\varepsilon} \nabla^2 v \, \mathrm{d}V = \int_{\partial\Omega_\varepsilon} \frac{\partial v}{\partial n} \mathrm{d}S. \tag{5}$$

On the other hand, we have that

$$\int_{\Omega_\varepsilon} \nabla^2 v \, \mathrm{d}V = \int_{\Omega_\varepsilon} \delta_P \, \mathrm{d}V = 1. \tag{6}$$

Note that the surface normal $\mathbf{n}$ points outwards by convention, hence it is parallel to $\mathbf{r}$, so that $\frac{\partial v}{\partial n} = \frac{\partial v}{\partial r} = \frac{A}{r}$. We can integrate over Γ by using polar coordinates again, noting that on the boundary we have that $r = \varepsilon$:

$$1 = \int_0^{2\pi} \frac{A}{\varepsilon} \varepsilon \mathrm{d}\theta = \int_0^{2\pi} A \mathrm{d}\theta = 2\pi A, \tag{7}$$

regardless of the choice for $\varepsilon > 0$. Hence, we have found that

$$v = \frac{1}{2\pi} \ln r. \tag{8}$$

2.1.2 Weak formulation

We set $f \equiv 0$. We multiply the original PDE with the fundamental solution and integrate it to obtain the weak formulation, using the Green-Gauss Theorem:

$$0 = \int_\Omega \nabla^2 u \, v \, \mathrm{d}V = \int_\Omega \nabla(\nabla u \, v) \mathrm{d}V - \int_\Omega \nabla v \nabla u \, \mathrm{d}V = \int_\Gamma \frac{\partial u}{\partial n} v \, \mathrm{d}S - \int_\Omega \nabla v \nabla u \, \mathrm{d}V. \tag{9}$$

Now we apply integration by parts on the last term, to make $\nabla^2 v$ appear, which equals the Dirac delta function by definition. This is helpful, since the convolution of Dirac delta with a function equals the function evaluated at the source point. We find that

$$0 = \int_\Gamma \frac{\partial u}{\partial n} v \, \mathrm{d}S - \int_\Gamma \frac{\partial v}{\partial n} u \, \mathrm{d}S + \int_\Omega u \nabla^2 v \, \mathrm{d}V = \int_\Gamma \frac{\partial u}{\partial n} v \, \mathrm{d}S - \int_\Gamma \frac{\partial v}{\partial n} u \, \mathrm{d}S + u(P). \tag{10}$$

Note that in the above we implicitly assumed that the Dirac delta is sourced at $P \in \Omega \setminus \Gamma$, since otherwise the last volume integral will not equal $u(P)$. Next, we consider the case where $P \in \Gamma$.

In case $P \in \Gamma$, we again consider a small circle C_ε centered around P with radius $\varepsilon > 0$. We denote by $C_\varepsilon^{\text{in}}$ the part of this circle that lies inside Ω, so that $C_\varepsilon = C_\varepsilon^{\text{in}} \cup (\Omega \setminus C_\varepsilon^{\text{in}}) =: C_\varepsilon^{\text{in}} \cup C_\varepsilon^{\text{out}}$. We denote the part of Γ that is inside C_ε by Γ_ε, and the circular boundary of C_ε by $\partial C_\varepsilon^{\text{in}} \cup \partial C_\varepsilon^{\text{out}}$. We consider the domain $\Omega \setminus C_\varepsilon^{\text{in}}$, with boundary $(\Gamma \setminus \Gamma_\varepsilon) \cup \partial C_\varepsilon^{\text{in}}$. See Figure 1. Observe that as $\varepsilon \downarrow 0$ this domain converges to Ω. By Green's second identity, we have in this domain that

$$0 = \int_{\Omega \setminus C_\varepsilon^{\text{in}}} \nabla^2 u \, v \, \mathrm{d}V = \int_{(\Gamma \setminus \Gamma_\varepsilon) \cup \partial C_\varepsilon^{\text{in}}} \frac{\partial u}{\partial n} v \, \mathrm{d}S - \int_{\Omega \setminus C_\varepsilon^{\text{in}}} \nabla v \nabla u \, \mathrm{d}V. \tag{11}$$

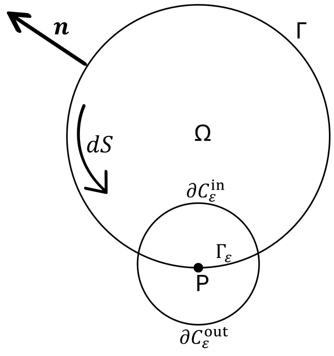

Figure 1: Geometry in the case that $P \in \Gamma$ and Ω is an interior domain.

Note that since P is not an element of the closure of $\Omega \setminus C_\varepsilon^{\text{in}}$, it holds that $\nabla^2 v = 0$ inside $\Omega \setminus C_\varepsilon^{\text{in}}$. We integrate by parts to get

$$0 = \int_{(\Gamma\setminus\Gamma_\varepsilon)\cup\partial C_\varepsilon^{\text{in}}} \frac{\partial u}{\partial n} v \, \mathrm{d}S - \int_{(\Gamma\setminus\Gamma_\varepsilon)\cup\partial C_\varepsilon^{\text{in}}} \frac{\partial v}{\partial n} u \, \mathrm{d}S + \int_{\Omega\setminus C_\varepsilon^{\text{in}}} u \nabla^2 v \, \mathrm{d}V, \tag{12}$$

where the last integral equals zero. We split the boundary integrals in the parts over $\Gamma\setminus\Gamma_\varepsilon$ (which converges to Γ) and $\partial C_\varepsilon^{\text{in}}$. We consider the latter part. Observe that, since the distance from P to any point on $\partial C_\varepsilon^{\text{in}}$ equals ε:

$$\int_{\partial C_\varepsilon^{\text{in}}} \frac{\partial u}{\partial n} v \, \mathrm{d}S = \int_{\partial C_\varepsilon^{\text{in}}} \frac{\partial u}{\partial n} \frac{1}{2\pi} \ln r \, \mathrm{d}S = \frac{\ln \varepsilon}{2\pi} \int_{\partial C_\varepsilon^{\text{in}}} \frac{\partial u}{\partial n} \mathrm{d}S. \tag{13}$$

For ε approaching zero, $\partial C_\varepsilon^{\text{in}}$ can be parametrized by an angle θ running from zero to π (i.e., $\partial C_\varepsilon^{\text{in}}$ is a semicircle). However, note that $\mathrm{d}S$ and $\mathrm{d}\theta$ are oriented in opposite directions, since both run counterclockwise (see Figure 1). We find

$$\int_{\partial C_\varepsilon^{\text{in}}} \frac{\partial u}{\partial n} \mathrm{d}S = -\int_0^\pi \frac{\partial u}{\partial n} r \, \mathrm{d}\theta = -\varepsilon \int_0^\pi \frac{\partial u}{\partial n} \, \mathrm{d}\theta. \tag{14}$$

Assuming $\frac{\partial u}{\partial n}$ is continuous (which is the case as ε approaches zero), we can apply the Mean Value Theorem to find a point $Q_\varepsilon \in \partial C_\varepsilon^{\text{in}}$ such that

$$\int_0^\pi \frac{\partial u}{\partial n} \mathrm{d}\theta = \left.\frac{\partial u}{\partial n}\right|_{Q_\varepsilon} \pi. \tag{15}$$

Hence, we conclude that

$$\int_{\partial C_\varepsilon^{\text{in}}} \frac{\partial u}{\partial n} v \, \mathrm{d}S = -\varepsilon \frac{\ln \varepsilon}{2\pi} \pi \left.\frac{\partial u}{\partial n}\right|_{Q_\varepsilon} \pi \xrightarrow{\varepsilon \downarrow 0} 0. \tag{16}$$

For the other boundary integral we have that

$$\int_{\partial C_\varepsilon^{\text{in}}} \frac{\partial v}{\partial n} u \, \mathrm{d}S = \int_{\partial C_\varepsilon^{\text{in}}} \frac{1}{2\pi r} u \, \mathrm{d}S = \frac{1}{2\pi\varepsilon} \int_{\partial C_\varepsilon^{\text{in}}} u \, \mathrm{d}S. \tag{17}$$

As ε approaches zero, we can again parametrize $\partial C_\varepsilon^{\text{in}}$ with an angle going from zero to π (oppositely oriented as $\mathrm{d}S$). We use this and the Mean Value Theorem as before to find that

$$\int_{\partial C_\varepsilon^{\text{in}}} \frac{\partial v}{\partial n} u \,\mathrm{d}S = -\frac{1}{2\pi\varepsilon}\int_0^\pi ur\,\mathrm{d}\theta = -\frac{1}{2\pi\varepsilon}u(Q_\varepsilon)\varepsilon\pi = -\frac{u(Q_\varepsilon)}{2}. \tag{18}$$

Since $Q_\varepsilon \in \partial C_\varepsilon^{\text{in}}$ with $\partial C_\varepsilon^{\text{in}}$ contracting to the point P as ε approaches zero, we get that

$$\int_{\partial C_\varepsilon^{\text{in}}} \frac{\partial v}{\partial n} u \,\mathrm{d}S \xrightarrow{\varepsilon\downarrow 0} -\frac{u(P)}{2}. \tag{19}$$

We combine the result of the two boundary integrals to conclude that for any point $P \in \Gamma$ it holds that

$$-\frac{u(P)}{2} = \int_\Gamma \frac{\partial u}{\partial n} v\,\mathrm{d}S - \int_\Gamma \frac{\partial v}{\partial n} u\,\mathrm{d}S, \tag{20}$$

because $\Gamma \setminus \Gamma_\varepsilon$ goes to Γ as ε approaches zero.

2.2 Exterior Laplace problem in 2D

In case Ω is not bounded, but instead the complement of a bounded domain, the boundary integral equation in (20) reads differently, due to changes in the geometry. The fundamental solution (the *freespace* Green's function) does not change, as it does not take the domain into account. Figure 2 shows the geometry in this case.

2.2.1 Weak formulation

Each of the integrals for $P \in \Gamma$ has the same limit as before, except the one that gave the $\frac{u(P)}{2}$ term. This integral now becomes

$$\int_{\partial C_\varepsilon^{\text{in}}} \frac{\partial v}{\partial n} u\,\mathrm{d}S = -\int_{\partial C_\varepsilon^{\text{in}}} \frac{1}{2\pi r} u\,\mathrm{d}S = -\frac{1}{2\pi\varepsilon}\int_{\partial C_\varepsilon^{\text{in}}} u\,\mathrm{d}S, \tag{21}$$

since now $\mathbf{n}$ and $\mathbf{r}$ (the vector connecting the point P to the point Q) point in opposite directions. We continue in a way analogous to the interior problem to find that, as ε approaches zero

$$\int_{\partial C_\varepsilon^{\text{in}}} u\,\mathrm{d}S = -\int_\pi^{2\pi} u\varepsilon\,\mathrm{d}\theta = -\pi\varepsilon u(Q_\varepsilon), \tag{22}$$

again by the Mean Value Theorem. Hence,

$$\int_{\partial C_\varepsilon^{\text{in}}} \frac{\partial v}{\partial n} u\,\mathrm{d}S \xrightarrow{\varepsilon\downarrow 0} \frac{u(P)}{2}, \tag{23}$$

so that

$$\frac{u(P)}{2} = \int_\Gamma \frac{\partial u}{\partial n} v\,\mathrm{d}S - \int_\Gamma \frac{\partial v}{\partial n} u\,\mathrm{d}S. \tag{24}$$

Note that the only difference with (20) is the minus sign on the left-hand side of the equation.

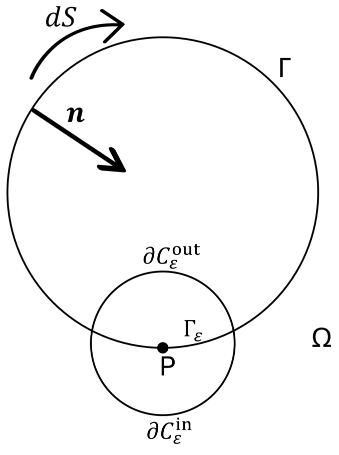

Figure 2: Geometry in the case that $P \in \Gamma$ and Ω is an exterior domain.

2.2.2 Approximation

Up until now, all derivations are exact. Our goal is to compute $\frac{\partial u}{\partial t}$: the tangential component of ∇u at the boundary Γ. We emphasize that $\frac{\partial}{\partial t}$ is **not** a time-derivative. To facilitate numerical computation of this quantity, we use so-called *constant boundary elements*: we discretize the boundary Γ into N line pieces Γ_i, along which u and $\frac{\partial u}{\partial n}$ are assumed to be constant. Figure 3 shows the discretized boundary. This leads to the following discretized equation for $u(P)/2$:

$$\frac{u(P)}{2} \approx \sum_{i=1}^{N}\left[\left(\frac{\partial u}{\partial n}\right)_i \int_{\Gamma_i} v \,\mathrm{d}S\right] - \sum_{i=1}^{N}\left[u_i \int_{\Gamma_i} \frac{\partial v}{\partial n}\mathrm{d}S\right]. \tag{25}$$

Observe that then

$$\frac{1}{2}\frac{\partial u}{\partial t} \approx \sum_{i=1}^{N}\left[\left(\frac{\partial u}{\partial n}\right)_i \frac{\partial}{\partial t}\left\{\int_{\Gamma_i} v \,\mathrm{d}S\right\}\right] - \sum_{i=1}^{N}\left[u_i \frac{\partial}{\partial t}\left\{\int_{\Gamma_i} \frac{\partial v}{\partial n}\,\mathrm{d}S\right\}\right]. \tag{26}$$

Since the Γ_i are fixed and do not depend on t, we can bring the partial derivative inside the integrals by the Leibniz rule. This yields

$$\frac{1}{2}\frac{\partial u}{\partial t} \approx \sum_{i=1}^{N}\left[\left(\frac{\partial u}{\partial n}\right)_i \int_{\Gamma_i} \frac{\partial v}{\partial t}\,\mathrm{d}S\right] - \sum_{i=1}^{N}\left[u_i \int_{\Gamma_i} \frac{\partial^2 v}{\partial n \partial t}\,\mathrm{d}S\right]. \tag{27}$$

We use the chain rule to compute the partial derivatives. Observe that

$$\frac{\partial v}{\partial t} = \frac{\partial v}{\partial r}\frac{\partial r}{\partial t} = \frac{1}{2\pi r}(-\sin\phi), \tag{28}$$

where we used the expression for $\frac{\partial r}{\partial t}$ as derived in Appendix A of the book by Katsikadelis [2]. Here, ϕ is the angle between the vector $\mathbf{r}$ (from the source point P to a point $q \in \Gamma_i$) and the surface normal $\mathbf{n}$.

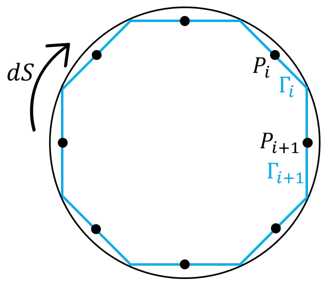

Figure 3: Discretized boundary Γ, with *constant* boundary elements Γ_i and nodal points P_i at the center of each Γ_i.

Similarly, as v is smooth:

$$\frac{\partial^2 v}{\partial n \partial t} = \frac{\partial}{\partial t}\left(\frac{1}{2\pi}\frac{\partial \ln r}{\partial r}\frac{\partial r}{\partial n}\right) = \frac{1}{2\pi}\frac{\partial}{\partial t}\left(\frac{1}{r}\frac{\partial r}{\partial n}\right) = \frac{1}{2\pi}\frac{\partial}{\partial t}\left(\frac{1}{r}\frac{\partial r}{\partial n}\right). \tag{29}$$

Appendix A of [2] derives that $\frac{\partial r}{\partial n} = \cos\phi$, $\frac{\partial r}{\partial t} = -\sin\phi$ and $\frac{\partial^2 r}{\partial n \partial t} = \frac{-\sin\phi\cos\phi}{r}$, which gives

$$2\pi\frac{\partial^2 v}{\partial n \partial t} = \frac{\partial r}{\partial n}\frac{\partial(1/r)}{\partial r}\frac{\partial r}{\partial t} + \frac{1}{r}\frac{\partial^2 r}{\partial n \partial t} = \frac{\cos\phi\sin\phi}{r^2} - \frac{\sin\phi\cos\phi}{r^2} = 0. \tag{30}$$

Apparently, the tangential component of ∇u on the boundary merely depends on the $\left(\frac{\partial u}{\partial n}\right)_i$ and not on the u_i. This means that we do not need to solve a linear system, but can directly compute the tangential components of the concentration gradient to the surface. We conclude that

$$\frac{1}{2}\frac{\partial u}{\partial t} \approx \sum_{i=1}^{N}\left[\left(\frac{\partial u}{\partial n}\right)_i \int_{\Gamma_i}\frac{-\sin\phi}{2\pi r}\mathrm{d}S\right] \implies \frac{\partial u}{\partial t} \approx -\frac{1}{\pi}\sum_{i=1}^{N}\left[\left(\frac{\partial u}{\partial n}\right)_i \int_{\Gamma_i}\frac{\sin\phi}{r}\mathrm{d}S\right]. \tag{31}$$

Now we appear to be in trouble, as the integral $\int_{\Gamma_i}\frac{\sin\phi}{r}dS$ is singular in case the point at which we aim to evaluate $\frac{\partial u}{\partial t}$ lies on Γ_i. Note that in this case $\sin\phi \in \{1, -1\}$, since the surface normal is perpendicular to $\mathbf{r}$ in this case along the entire constant boundary element Γ_i. We return to this case later.

We compute the tangential component of the gradient of u at the center P_i of each boundary element Γ_i. We then need to solve

$$\left(\frac{\partial u}{\partial t}\right)_i = -\frac{1}{\pi}\sum_{j=1}^{N}\left[\left(\frac{\partial u}{\partial n}\right)_j \int_{\Gamma_j}\frac{\sin\phi_i}{r_i}\mathrm{d}S\right] \quad \forall i = 1, \ldots, N, \tag{32}$$

where r_i is the distance from P_i to the running coordinate over the boundary element and ϕ_i the angle between $\mathbf{r}_i$ and $\mathbf{n}$. This system of equations can be written in matrix form as

$$\mathbf{u}_t = -\frac{1}{\pi}\mathbf{H}\mathbf{u}_n, \tag{33}$$

where

$$\mathbf{u}_t := \left(\left(\frac{\partial u}{\partial t}\right)_1, \ldots, \left(\frac{\partial u}{\partial t}\right)_N\right)^T, \qquad \mathbf{u}_n := \left(\left(\frac{\partial u}{\partial n}\right)_1, \ldots, \left(\frac{\partial u}{\partial n}\right)_N\right)^T,$$
$$\mathbf{H} = (h_{ij})_{1\leq i,j\leq N} := \left(\int_{\Gamma_j} \frac{\sin\phi_i}{r_i} \mathrm{d}S\right)_{1\leq i,j\leq N}. \tag{34}$$

2.2.3 Computing the matrix elements

First, we aim to compute u. We need

$$a_{ij} = \int_{\Gamma_j} \frac{\ln(r_i)}{2\pi}\mathrm{d}S = \int_{-1}^{1} \frac{\ln\left(\sqrt{(x_j^+ + x_j^-\xi - p_x)^2 + (y_j^+ + y_j^-\xi - p_y)^2}\right)}{2\pi}\frac{l_j}{2}\mathrm{d}\xi, \tag{35}$$

and

$$b_{ij} = \int_{\Gamma_j} \frac{1}{2\pi r_i}\cos\phi_i \mathrm{d}S \tag{36}$$

according to Appendix A of [2].

The goal is now to compute the matrix elements h_{ij}. To that end, we should express $\sin\phi_i$ in known quantities. Appendix A of [2] gives us that

$$\mathbf{n} = (\cos\beta_i, \sin\beta_i)^T, \qquad \mathbf{q} - \mathbf{P}_i = r_i(\cos\alpha_i, \sin\alpha_i)^T, \tag{37}$$

where q is the running point over Γ_j in the integral, and $\phi = \beta - \alpha$. Then we can use that

$$\sin\phi_i = \cos\alpha\sin\beta - \sin\alpha\cos\beta = \frac{1}{r_i}\left[(q_x - p_x)n_y - (q_y - p_y)n_x\right], \tag{38}$$

where $\mathbf{n} = (n_x, n_y)^T = -(q_x, q_y)^T/\sqrt{q_x^2 + q_y^2}$, $q = (q_x, q_y)$ and $P_i = (p_x, p_y)$. To evaluate the line integrals over the boundary elements, we transform the global coordinates (x, y) to local coordinates (x', y') (where x' runs parallel to the boundary element and y' perpendicular), following Chapter 3 of [2]. However, note that the coordinates along the boundary elements in our case run in a direction opposite to that of [2]. Let l_j denote the length of boundary element Γ_j, and (x_j, y_j) the extreme point on both Γ_j and Γ_{j-1}, then

$$(x, y) = \left(\frac{x_{j+1} + x_j}{2} + \frac{x_{j+1} - x_j}{l_j}x', \frac{y_{j+1} + y_j}{2} + \frac{y_{j+1} - y_j}{l_j}x'\right), \quad -\frac{l_j}{2} \leq x' \leq \frac{l_j}{2}. \tag{39}$$

Introducing the dimensionless coordinate $\xi := \frac{x'}{l_j/2}$ gives

$$(x(\xi), y(\xi)) = \left(\frac{x_{j+1} + x_j}{2} + \frac{x_{j+1} - x_j}{2}\xi, \frac{y_{j+1} + y_j}{2} + \frac{y_{j+1} - y_j}{2}\xi\right), \quad -1 \leq \xi \leq 1. \tag{40}$$

It then readily follows that in the integrals

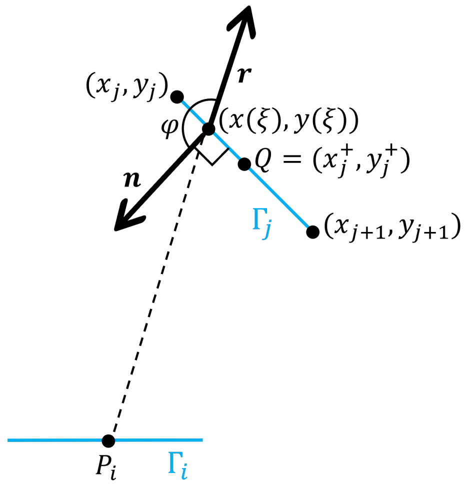

Figure 4: Geometry of a boundary element in case $i \neq j$.

$$dS = \sqrt{(dx)^2 + (dy)^2} = \sqrt{\frac{(x_{j+1} - x_j)^2}{4}(d\xi)^2 + \frac{(y_{j+1} - y_j)^2}{4}(d\xi)^2} = \sqrt{\left|\begin{pmatrix} x_{j+1} \\ y_{j+1} \end{pmatrix} - \begin{pmatrix} x_j \\ y_j \end{pmatrix}\right|^2} \frac{d\xi}{2} = \frac{l_j}{2} d\xi. \quad (41)$$

We note that for constant boundary elements $\mathbf{n} = -\hat{\mathbf{q}} = -(q_x, q_y)^T / \sqrt{q_x^2 + q_y^2}$ is constant along the boundary element Γ_j with central node $Q = (x_j^+, y_j^+)$. See Figure 4 for a schematic of the geometry. We get

$$h_{ij} = \int_{-1}^{1} \frac{\sin \phi_i}{r_i(\xi)} \frac{l_j}{2} d\xi = \int_{-1}^{1} \frac{(x(\xi) - p_x)n_y - (y(\xi) - p_y)n_x}{r_i^2(\xi)} \frac{l_j}{2} d\xi = \frac{l_j}{2} \int_{-1}^{1} \frac{\begin{pmatrix} x(\xi) - p_x \\ y(\xi) - p_y \end{pmatrix} \times (-\hat{\mathbf{q}})}{r_i^2(\xi)} d\xi, \quad (42)$$

where $\begin{pmatrix} a \\ b \end{pmatrix} \times \begin{pmatrix} c \\ d \end{pmatrix}$ is defined as $ad - bc$, and $r_i(\xi) = \sqrt{(x(\xi) - p_x)^2 + (y(\xi) - p_y)^2}$. This integral can be evaluated exactly for $i \neq j$. We denote $x_j^+ = \frac{x_{j+1} + x_j}{2}$, $y_j^+ = \frac{y_{j+1} + y_j}{2}$, $x_j^- = \frac{x_{j+1} - x_j}{2}$, $y_j^- = \frac{y_{j+1} - y_j}{2}$ and find

$$h_{ij} = \frac{l_j}{2} \int_{-1}^{1} \frac{-(x_j^+ + x_j^- \xi - p_x)y_j^+ + (y_j^+ + y_j^- \xi - p_y)x_j^+}{\sqrt{(x_j^+)^2 + (y_j^+)^2}\left[(x_j^+ + x_j^- \xi - p_x)^2 + (y_j^+ + y_j^- \xi - p_y)^2\right]} d\xi. \quad (43)$$

We take out the constant square in the denominator and focus on the integral

$$\int_{-1}^{1} \frac{(y_j^- x_j^+ - x_j^- y_j^+)\xi + (y_j^+ - p_y)x_j^+ - (x_j^+ - p_x)y_j^+}{((x_j^-)^2 + (y_j^-)^2)\xi^2 + (2x_j^-(x_j^+ - p_x) + 2y_j^-(y_j^+ - p_y))\xi + (x_j^+ - p_x)^2 + (y_j^+ - p_y)^2} d\xi =: I. \quad (44)$$

We gather all the constants and write

$$I =: \int_{-1}^{1} \frac{a\xi + b}{A\xi^2 + B\xi + C} d\xi = \frac{a}{2A} \int_{-1}^{1} \frac{2A\xi + B}{A\xi^2 + B\xi + C} d\xi + \int_{-1}^{1} \frac{b - \frac{aB}{2A}}{A\xi^2 + B\xi + C} d\xi =: I_1 + I_2. \quad (45)$$

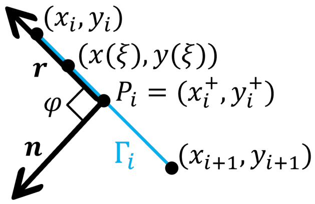

Figure 5: Geometry of a boundary element in case $i = j$.

The first integral is readily evaluated using the substitution $u = A\xi^2 + B\xi + C$:

$$I_1 = \frac{a}{2A}\int_{A-B+C}^{A+B+C} \frac{\mathrm{d}u}{u} = \frac{a}{2A}(\ln|A+B+C| - \ln|A-B+C|). \tag{46}$$

For the second integral we find

$$I_2 = \frac{b - \frac{aB}{2A}}{A}\int_{-1}^{1} \frac{1}{\left(\xi + \frac{B}{2A}\right)^2 + \frac{C}{A} - \frac{B^2}{4A^2}}\mathrm{d}\xi = \frac{b - \frac{aB}{2A}}{A\left(\frac{C}{A} - \frac{B^2}{4A^2}\right)}\int_{-1}^{1} \frac{1}{\left(\frac{\xi + \frac{B}{2A}}{\sqrt{\frac{C}{A} - \frac{B^2}{4A^2}}}\right)^2 + 1}\mathrm{d}\xi. \tag{47}$$

We use the substitution $v(\xi) = \frac{\xi + B/(2A)}{\sqrt{C/A - B^2/(4A^2)}}$ to obtain

$$I_2 = \frac{b - \frac{aB}{2A}}{A\sqrt{\frac{C}{A} - \frac{B^2}{4A^2}}}\int_{v(-1)}^{v(1)} \frac{1}{v^2+1}\mathrm{d}v = \frac{b - \frac{aB}{2A}}{A\sqrt{\frac{C}{A} - \frac{B^2}{4A^2}}}(\arctan(v(1)) - \arctan(v(-1))). \tag{48}$$

Recall that

$$h_{ij} = \frac{l_j}{2\sqrt{(x_j^+)^2 + (y_j^+)^2}}(I_1 + I_2), \quad i \neq j, \tag{49}$$

where I_1, I_2 depend on both i and j.

Finally, we return to the case where $i = j$, i.e., where $(p_x, p_y) = (x_i^+, y_i^+)$. See Figure 5 for a schematic of the geometry in this case. Note that here $\mathbf{r}$ and $\mathbf{n}$ are at an angle $\pi/2$ in the first half of Γ_i and at an angle $3\pi/2$ in the second half, so that

$$h_{ii} = \int_{-1}^{0} \frac{1}{r_i(\xi)}\frac{l_i}{2}\mathrm{d}\xi + \int_{0}^{1} \frac{-1}{r_i(\xi)}\frac{l_i}{2}\mathrm{d}\xi = \frac{l_i}{2}\left[\int_{0}^{-1} \frac{-1}{r_i(\xi)}\mathrm{d}\xi + \int_{0}^{1} \frac{-1}{r_i(\xi)}\mathrm{d}\xi\right]. \tag{50}$$

Now we substitute $\hat{\xi} = -\xi$ and $d\xi = -d\hat{\xi}$ in the first integral to find that

$$h_{ii} = \frac{l_i}{2}\left[\int_{0}^{1} \frac{1}{r_i(\hat{\xi})}\mathrm{d}\hat{\xi} + \int_{0}^{1} \frac{-1}{r_i(\xi)}\mathrm{d}\xi\right] = 0, \tag{51}$$

as $r_i(\xi) = \sqrt{(x_i^- \xi)^2 + (y_i^- \xi)^2} = \sqrt{(x_i^-)^2 + (y_i^-)^2}|\xi| = r_i(-\xi)$, in case $i = j$.

We can now find the magnitude of the tangential component of the gradient at the center of each boundary element, but we need its direction too. This is simply the direction of the running coordinate ξ, and can be computed as

$$\mathbf{t} = \begin{pmatrix} x_{j+1} - x_j \\ y_{j+1} - y_j \end{pmatrix} / \sqrt{(x_{j+1} - x_j)^2 + (y_{j+1} - y_j)^2}, \tag{52}$$

for boundary element Γ_j.

2.3 2D Exterior Rod

Since we do not have a smooth surface anymore in case of a rod, we may have to carefully consider the sharp corners. For $P \in \Gamma$ **not** a corner point:

$$\frac{u(P)}{2} = \int_\Gamma \frac{\partial u}{\partial n} v \mathrm{d}S - \int_\Gamma \frac{\partial v}{\partial n} u \mathrm{d}S \approx \sum_{i=1}^{N} \left[\left(\frac{\partial u}{\partial n} \right)_i \int_{\Gamma_i} v \mathrm{d}S \right] - \sum_{i=1}^{N} \left[u_i \int_{\Gamma_i} \frac{\partial v}{\partial n} \mathrm{d}S \right].$$

For $P \in \Gamma$ a corner point:

$$\frac{u(P)}{4} = \int_\Gamma \frac{\partial u}{\partial n} v \mathrm{d}S - \int_\Gamma \frac{\partial v}{\partial n} u \mathrm{d}S \approx \sum_{i=1}^{N} \left[\left(\frac{\partial u}{\partial n} \right)_i \int_{\Gamma_i} v \mathrm{d}S \right] - \sum_{i=1}^{N} \left[u_i \int_{\Gamma_i} \frac{\partial v}{\partial n} \mathrm{d}S \right].$$

However, we do not really care about corner points, since we compute $\frac{\partial u}{\partial t}$ at the *center* of each Γ_i. We can thus proceed exactly as before, but with differently oriented boundary elements. The details are omitted, but the code implementing the derivations is included. For the Python code implementing the discretized boundary integral equation to find the 2D rod-shaped micromotor's propulsion speed, see `https://github.com/Maurik-24/BEM_micromotors`.

The pseudocode in the below algorithm details how the simulated propulsion speed and the diffusion parameters fitted from experimental data are used to simulate micromotor trajectories and thus Mean Squared Displacement (MSD) profiles.

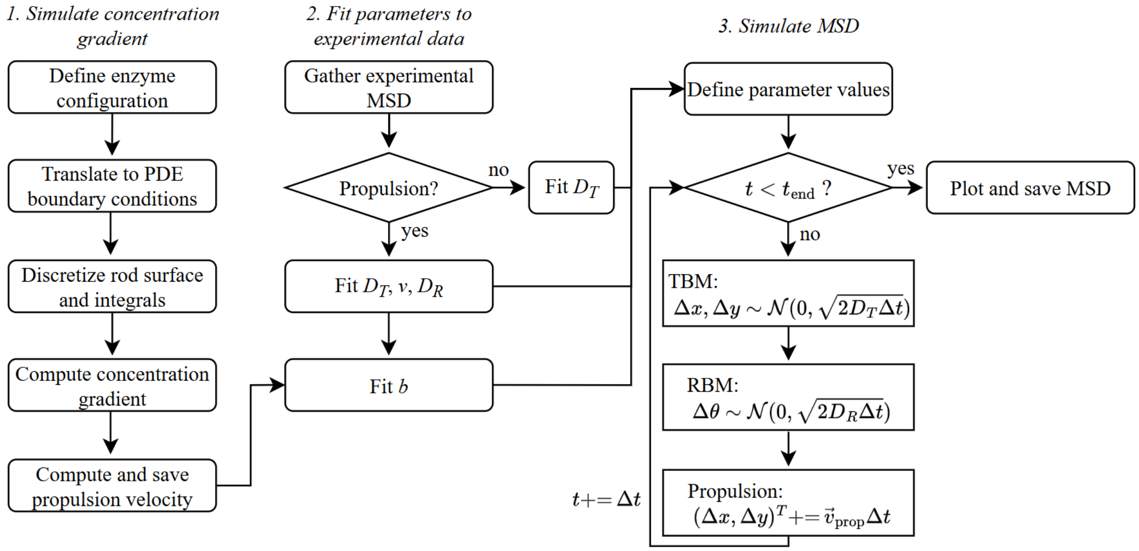

Algorithm 1. Pseudocode for MSD simulation. TBM and RBM are translational and rotational Brownian motion, respectively. MSD profiles are gathered from many trajectories simulated as in the time loop.

3 References

[1] W. Dijkstra. *Condition numbers in the boundary element method: shape and solvability*. PhD thesis, Eindhoven University of Technology, 2008.

[2] John T Katsikadelis. *The boundary element method for engineers and scientists: theory and applications*. Academic Press, 2016.

[3] Mihail N Popescu, William E Uspal, and Siegfried Dietrich. Self-diffusiophoresis of chemically active colloids. *The European Physical Journal Special Topics*, 225:2189–2206, 2016.
